# Supramolecular Polymer‐Surfactant Co‐Assemblies for Multivalent HSV‐1 Inhibition

**DOI:** 10.1002/anie.8702628

**Published:** 2026-07-26

**Authors:** Christian Zoister, Boris Schade, Paulina Sittinger, Xianwen Lou, Esmee de Korver, Freek V. de Graaf, Ranen Etouki, Chuanxiong Nie, Kai Licha, Kai Ludwig, Abhishek Kumar Singh, E. W. Meijer, Rainer Haag

**Affiliations:** ^1^ Institute of Chemistry and Biochemistry Freie Universität Berlin Berlin Germany; ^2^ Institute for Complex Molecular Systems, Laboratory of Macromolecular and Organic Chemistry Eindhoven University of Technology Eindhoven The Netherlands; ^3^ Forschungszentrum für Elektronenmikroskopie, Core‐Facility BioSupraMol, Institute of Chemistry and Biochemistry Freie Universität Berlin Berlin Germany; ^4^ School of Biosciences Chanakya University Bengaluru India; ^5^ School of Chemistry and RNA Institute University of New South Wales Sydney New South Wales Australia; ^6^ Max Planck Institute for Polymer Research Mainz Germany

**Keywords:** co‐assembly, multivalent, nanofiber, supramolecular, virus

## Abstract

Supramolecular assemblies are of fundamental importance for cellular life and bio‐interactive materials. The structure and function of these assemblies are decisively influenced by the design of their molecular components. In this work, we present a modular synthetic strategy for six novel bio‐functional surfactants with a sulfonate headgroup (termed *SupraVir* surfactants). These amphiphilic molecules were designed for strong interactions with viral envelope proteins, virus inhibition, and to co‐assemble water‐soluble benzene‐1,3,5‐tricarboxamide derivatives (BTAs) to generate surfactant‐grafted supramolecular polymers. Systematic spectroscopic and microscopic studies showed that the morphology of the co‐assemblies strongly depends on the surfactant‐BTA ratio. At surfactant ratios higher than 1, the native BTA nanofibers were disrupted, resulting in micellar architectures. In the test with herpes simplex virus (HSV‐1), fibrous supramolecular polymers show significantly lower half‐maximal inhibitory concentrations (IC_50_) compared to the corresponding micellar aggregates of the pure surfactants. Interestingly, the co‐assemblies exhibited lower cytotoxicity than the functional surfactants alone, probably due to their structured incorporation into the polymeric scaffold. The dynamics of the co‐assemblies, probed by hydrogen‐deuterium exchange mass spectrometry (HDX‐MS), showed correlations to the effectiveness. These results demonstrate a new co‐assembly strategy of functional positioning antiviral surfactants in supramolecular scaffolds for the development of multivalent antiviral materials.

## Graphical abstract

Non‐covalent co‐assembly of **BTA** with antiviral surfactants produces ratio‐dependent morphologies. Fibrous supramolecular polymers exhibit superior HSV‐1 inhibition, revealing multivalent fiber‐virus interactions and highlighting supramolecular co‐assemblies as a powerful strategy for functional biomedical nanomaterials with demonstrated tunable structure‐activity relationships.

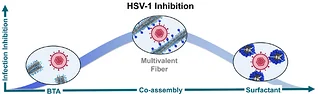

## 1 Introduction

In nature, noncovalent interactions enable the formation of highly dynamic and functional supramolecular structures [1, 2, 3]. Supramolecular assemblies and their self‐organization are of fundamental importance for biological organization and function. These structures can be observed in protein folding, lipid membranes, and viral envelopes, to name a few [4, 5, 6]. Complex self‐organized formation of functional assemblies in short time regimes is subject of current research [4]. It is well known that non‐covalent forces, like hydrophobic interactions, hydrogen bonds, and other weak interactions, play a key role in these processes [7]. Supramolecular polymers are especially important in nature and are of high interest for bio‐inspired advanced materials because of their combination of structure, function, and dynamics [8]. For this reason, scientists are researching synthetic supramolecular polymers for biomedical applications, including drug delivery and antiviral therapies [9, 10, 11].

Next to supramolecular polymers, small‐molecule amphiphiles (surfactants) are effective monomeric building blocks for self‐assembly. Surfactants can be synthesized and modified in a modular fashion, facilitating the development of biocompatible systems with well‐defined architecture. Functional surfactants offer tunable amphiphilicity, multivalent display of active groups, and show interesting self‐assembly potential [12, 13]. Easily accessible surfactants with different structural motifs can be used as additives for protein folding, or for cellular recognition, membrane protein purification, and drug delivery [14, 15, 16]. Oligoglycerol‐based surfactants have been extensively investigated and offer distinct advantages. These surfactants have multiple terminal hydroxyl groups, making them highly suitable for post‐functionalization with various active groups for targeted applications [17, 18, 19]. Due to their structural versatility and increasing therapeutic relevance, the Haag group has investigated a wide range of such surfactants. Different generations of oligoglycerols and a variety of hydrophobic segments have been used. This includes alkyl and fluoroalkyl chains to improve their functionality and applicability in various biomedical fields [10, 17, 20].

Moreover, Stupp and coworkers have presented supramolecular peptide‐based amphiphiles assembling into nanofibers, which can inhibit the cell entry of SARS‐CoV‐2 pseudovirus [11]. Directed hydrogen‐bonding in peptide amphiphiles ensures stable and relatively stiff nanofibers, although their dynamic properties are recently highlighted [21, 22]. Surfactants provide useful means of influencing assemblies’ structure and dynamics, while covalent polymers contribute stability and defined architectures. Building on these strengths, supramolecular polymers offer a distinctive advantage: their dynamic and structurally flexible nature enables them to effectively adapt to the viral morphology [23, 24].

The self‐assembly of various benzene‐1,3,5‐tricarboxamide monomers (**BTA**s) into supramolecular polymers has been extensively studied [25, 26]. In aqueous solution, directed hydrogen bonding, the hydrophobic effect, and pi‐pi stacking between the monomers lead to formation of helical structures. Functional **BTA**s have been developed for various biomedical applications, including bacterial inhibition and intracellular delivery [27, 28]. This requires the synthetic modification of **BTA**s to introduce novel functionalities into copolymer assemblies. Amphiphilic structures, such as surfactants, can also be incorporated into **BTA**s to form supramolecular nanostructures, providing a toolbox for developing **BTA**‐based functional biomaterials. Su et al. reported gel‐sol‐gel‐sol transitions in **BTA** mixtures caused by morphological changes in nanofibers due to surfactant addition [29]. Recently, we reported on amplifying asymmetry through structural transitions in **BTA**‐surfactant systems. Using easily accessible, chiral, non‐ionic oligoglycerol surfactants results in a preferred helicity of the co‐assembled polymers. This highlights the possible impact of surfactants on the overall properties of supramolecular assemblies and their subsequent effect in bio‐functional applications [30]. The observed asymmetry transfer from chiral surfactants to supramolecular fibers raises the question of its applicability to other fields, such as bio‐functional applications. The co‐assembly of functional surfactants with **BTAs** can reduce the intrinsic cytotoxicity of the surfactants themselves by arranging them spatially within the supramolecular polymeric scaffold. Additionally, fibrous architecture can promote multivalent interactions with viruses, thereby increasing antiviral efficacy.

Our group has previously reported on two‐dimensional nano‐sheets derived from poly‐glycerol polymers that were pre‐modified to interact with viral particles, leading to shielding and subsequent inhibition of the virus [31]. Furthermore, we have explored two‐dimensional nanodiscs and three‐dimensional liposome‐like co‐assemblies formed via charge‐driven self‐assembly [32]. While these approaches focused on the pre‐modification of two‐ and three‐dimensional architectures for virus inhibition, the current work shifts the focus to one‐dimensional noncovalent co‐assemblies, offering a unique design strategy and mechanistic framework.

**FIGURE 1:**
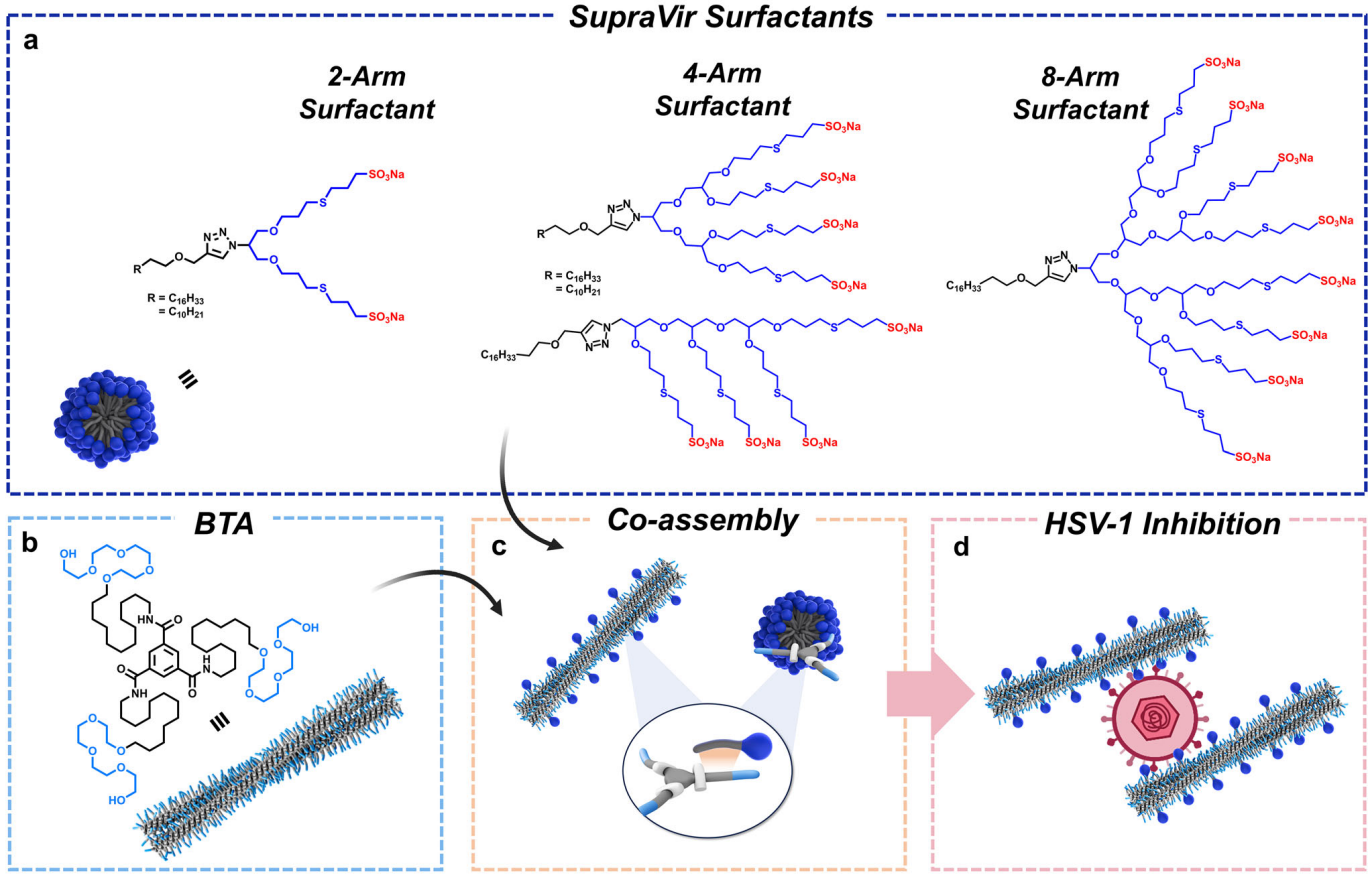
(a) Novel *SupraVir* surfactants that were synthesized for effective interaction with and inhibition of HSV‐1. The surfactants are arranged from left to right in order of increasing headgroup size and hydrophilicity. The surfactants self‐assemble into small spherical micelles. (b) The molecular structure of benzene tricarboxyamides (**BTA**) and their self‐assembly into racemic double helical fibers in an aqueous solution. (c) Co‐assembly formation by varying the ratio of **BTA** to surfactant. (d) Investigation of biocompatibility and assessment of antiviral activity, which results in improved HSV‐1 inhibition and cell viability for co‐assemblies in fibrous assembly.

In this study, we tested supramolecular **BTA**‐surfactant co‐assemblies for inhibiting herpes simplex virus (HSV‐1). We synthesized a series of antiviral surfactants (Figure 1a) that differ in alkyl chain length and degree of hydrophilicity. We investigated their co‐assemblies with the supramolecular **BTA** scaffold and visualized ratio‐dependent morphological transitions. Evaluation of the biocompatibility and antiviral activity of the co‐assemblies revealed increased efficiency against the virus, while the cytotoxicity of the surfactants was significantly reduced. These new insights provide valuable design principles for the development of novel, multivalent antiviral materials based on easily accessible functional surfactants intercalated in supramolecular polymers.

## 2 Results and Discussion

### 2.1 Design and Synthesis of the Surfactants

We designed the surfactant synthesis using a modular approach and two types of click chemistry to combine the hydrophobic and hydrophilic parts of the surfactants. These accessible click reaction conditions enabled us to easily generate a series of surfactants.

To elucidate the structure–activity relationships (SAR) relevant to antiviral supramolecular systems, we designed a series of surfactants that differ in alkyl chain length (C_12_ and C_18_) and headgroup architecture (2‐, 4‐, and 8‐branched structures). These branched architectures are obtained by post‐functionalizing an oligoglycerol core with sulfonate groups to introduce negative charges that interact with the virus while enabling good water solubility. Systematic variation of the headgroup topology allowed us to investigate how molecular branching affects assembly behavior with n**BTA** and the resulting supramolecular structures. For the eight‐armed surfactant, we introduced a longer C_18_ alkyl chain to offset the increased hydrophilicity of the highly branched headgroup. To further explore the role of molecular geometry, we synthesized a linear analogue of the **C_18_‐4S** surfactant, hereinafter referred to as **C_18_‐lTG‐4S** (a derivative of a surfactant based on a linear triglycerol headgroup). In this surfactant, the alkyl chain is attached to a primary hydroxy group, in contrast to the secondary position in the dendritic derivative. The idea was to investigate whether the linear headgroup displays its functionality differently from the dendritic **C_18_‐4S**. This series of surfactants provides a modular platform to better understand how structural features control co‐assembly and to identify key molecular parameters that influence antiviral performance.

All monomers were synthesized under conditions previously used for our cationic surfactants, as described in the literature [19]. The dendritic oligoglycerol units established by Wyszogrodzka et al. were used as the hydrophilic headgroups [33]. Scheme S1 illustrates the synthesis of the dendritic four‐arm surfactant. The protected first‐generation dendritic oligo‐glycerol (pG1‐OH) was mesylated and converted into an azide. The acetal‐protected hydroxyl groups were deprotected using acidic polystyrene resin (Dowex‐50WX8). To enable thiol‐ene click reactions, the hydroxyl groups underwent Williamson ether synthesis with allyl bromide to be alkylated. Cu(I)‐catalyzed azide–alkyne cycloaddition (CuAAC) was then used to link the alkyl chain to the oligo‐glycerol headgroup, forming a 1,4‐triazole linker. The copper‐catalyzed click reactions were performed in good yields (>85%). The characteristic triazole peaks in the ^1^H‐NMRs (supporting information), between 7–8 ppm, confirmed product formation. Lastly, thiol‐ene click chemistry between the allyl groups and sodium 3‐mercaptopropyl sulfonate introduced sulfonates to the surfactants’ heads. Once again, click chemistry led to good reaction yields under UV‐light in short reaction times of only a few hours.

**SCHEME 1:**
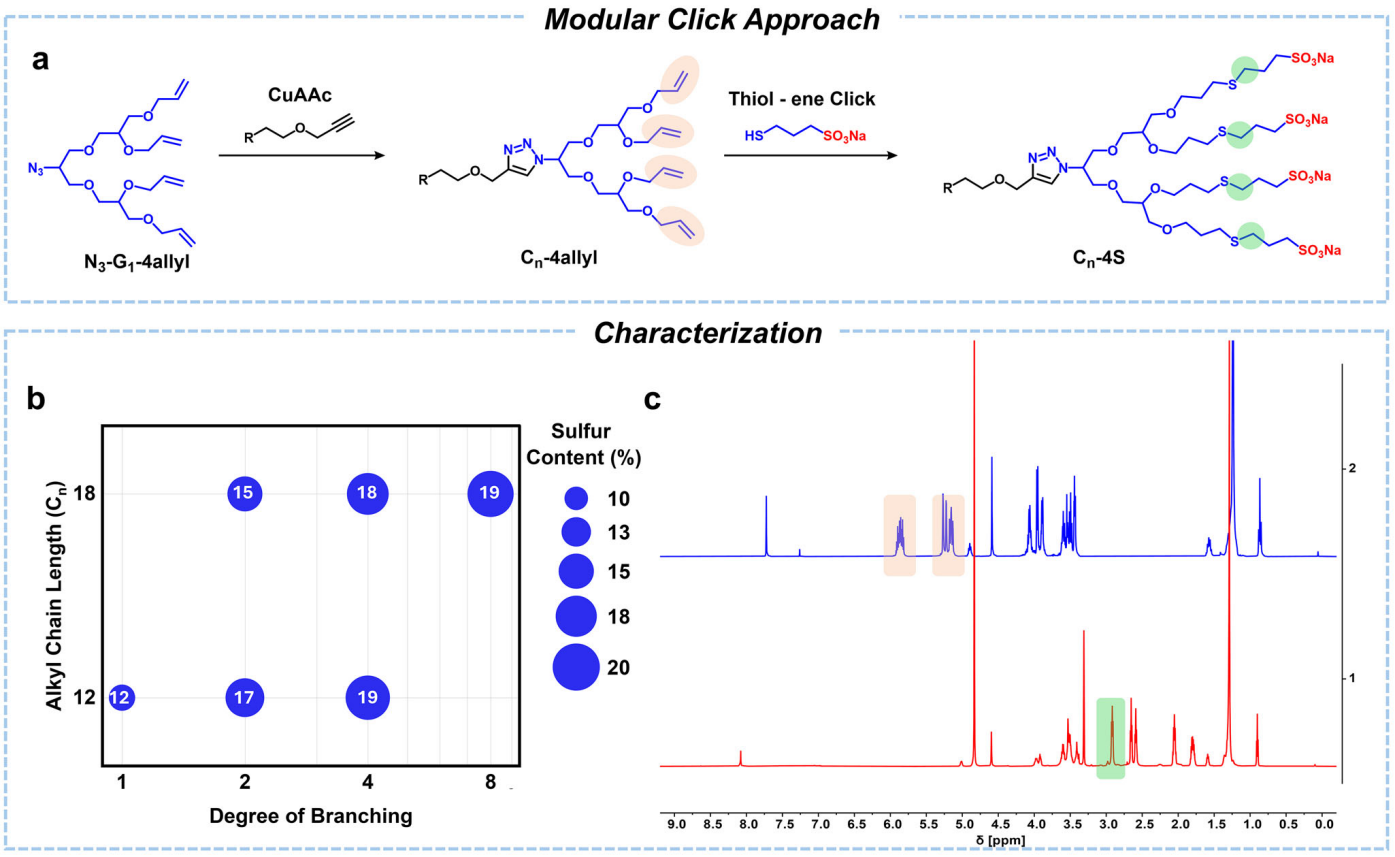
(a) Dual click‐chemistry for modular synthesis of sulfonated *SupraVir* surfactants. R = C_10_H_21_ or C_16_H_33_. First, Cu(I)‐catalyzed azide–alkyne cycloaddition (CuAAC) is used to introduce the alkyl chain. Then the final thiol‐ene click reaction introduces sulfonates. (b) Characterization of *SupraVir* surfactants by elemental analysis, to visualize the increasing sulfur content, and therefore hydrophilicity, in correlation to alkyl chain length and degree of branching. Size of circles correlates to the sulfur content in percent. (c) A ^1^H‐NMR comparison between C_18_‐4allyl (top) and **C_18_‐4S** (bottom) shows the disappearance of the allyl protons (5–6 ppm, orange) after the final thiol‐ene click reaction.

The final surfactants lack the characteristic allyl peaks (5–6 ppm) proving full reaction conversion, as exemplified with **C_18_‐4S** in Scheme 1c. All structures were characterized using ^1^H and ^13^C NMR spectroscopy and mass‐spectrometry. With an higher degree of branching (Figure 1a, from left to right), the number of sulfonates increases and thereby the hydrophilicity. This increase in hydrophilicity is visualized by the sulfur content of the surfactants, which is presented in correlation to the length of the alkyl chains and the degree of branching in Scheme 1b.

### 2.2 Physicochemical Characterization

Surfactants or small‐molecule amphiphiles exhibit unique physicochemical properties in aqueous solutions. We studied their aggregation behavior using spectroscopy and dynamic light scattering (DLS); the results are combined in Table S2. The measurements were performed in phosphate‐buffered saline (PBS) to simulate physiological conditions. The presence of ions is known to influence the aggregation behavior of surfactants, typically reducing head‐to‐head repulsion and leading to lower critical micelle concentrations (CMCs) [34]. Therefore, it is important to simulate the conditions used in subsequent cell and virus studies. DLS results (Figure S1) show particle sizes between 5 and 18 nm for C_18_ surfactants. C_12_ surfactants seem to form larger aggregates, ranging from 110 to 370 nm, as seen with the commercially available sodium dodecyl sulfate (**SDS**). The zeta potentials of all *SupraVir* surfactants range from −40 to −50 mV (Table S2), depending on the pH. These values are kept constant by a diluted PBS buffer [35]. All surfactants form strong, stable, negatively charged micelles and the CMCs derived from the DLS count rate show a consistent trend for all synthesized surfactants (Figure S2). Longer alkyl chains (C_18_) result in lower CMCs, as shown by **C_18_‐2S** (6 µM) compared with **C_12_‐2S** (100 µM) [36, 37]. On the other hand, larger ionic headgroups increase solubility and lead to higher CMCs, as seen with **C_18_‐4S** (40 µM) and **C_18_‐8S** (80 µM) [38]. Notably, **SDS** shows a much higher CMC (1350 µM), which can be explained by the headgroup's structural difference, leading to linear morphology that strongly impacts micelle formation [34]. In conclusion, all synthesized surfactants containing sulfonates in their headgroups form small spherical micelles in aqueous solution.

### 2.3 Co‐assembly With BTA Nanofiber

The combination of **BTA** supramolecular polymers and surfactants enables the formation of co‐assemblies that lead to aggregates with unique physicochemical properties that differ from those of the individual components [30]. These structural and dynamic properties were investigated by spectroscopy, microscopy, and mass‐spectrometry. Stock solutions of **BTA** and surfactants were prepared by dissolving the solid compounds in water, heating the mixture for 15 min at 80°C, and then vortexing vigorously. These solutions were mixed in different stoichiometric ratios and assembled using the precise heating and equilibration protocol described in the method section (SI). Heating to 80°C followed by cooling to room temperature, establishes a new equilibrium at which the surfactants are incorporated into the **BTA** nanofibers [29].

**FIGURE 2:**
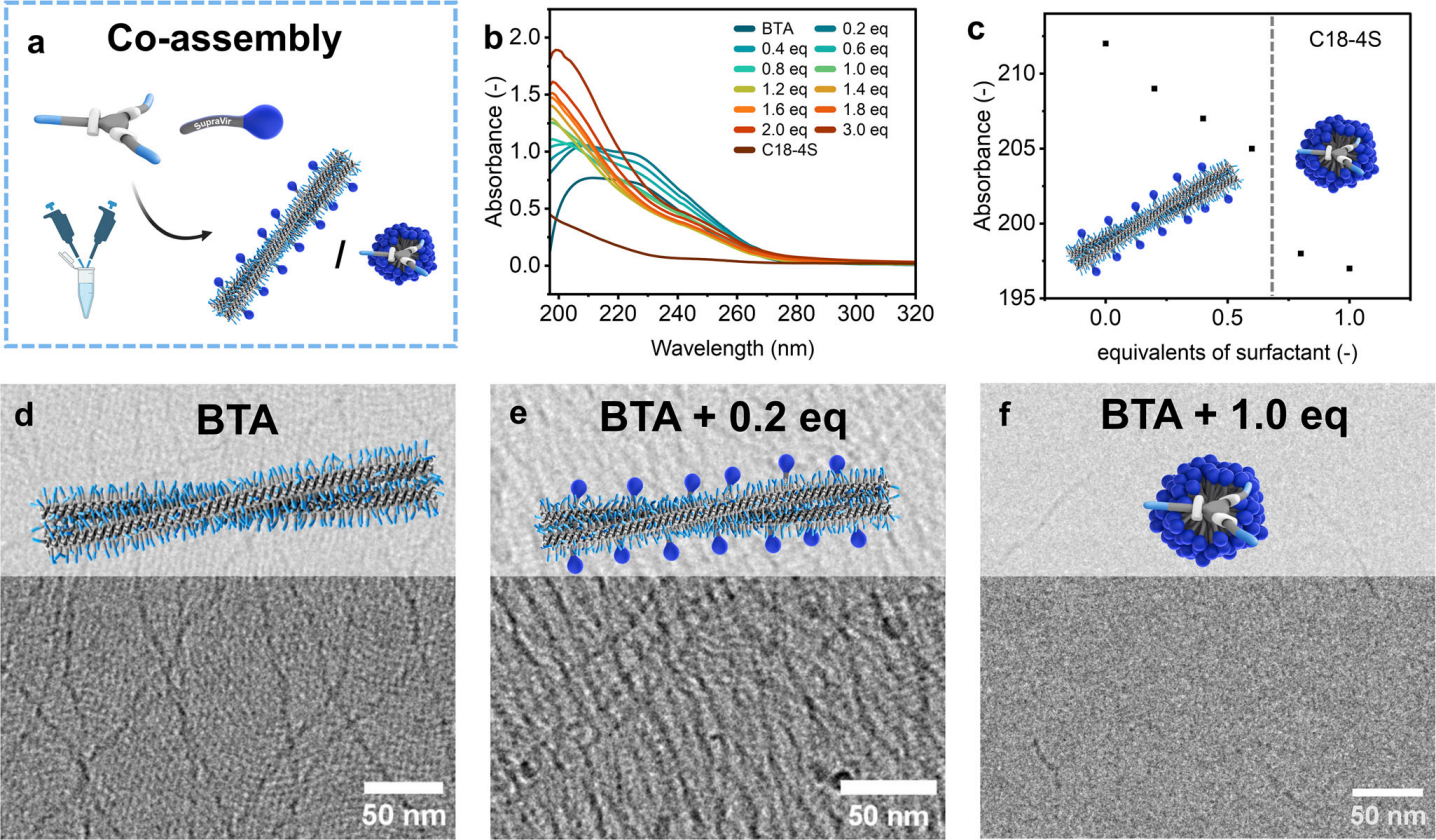
Typical **BTA** co‐assemblies with a surfactant; here, **C_18_‐4S** is used as an example. (a) Preparation scheme of the co‐assemblies using **BTA** and surfactant stock solutions prepared according to a precise heating and equilibration protocol (SI). (b) UV‐vis spectra of **BTA** (250 µM) with increasing molar equivalents of **C_18_‐4S** visualize a morphological change with the addition of the surfactant. (c) Wavelengths of the absorbance maxima plotted against the amount of **C_18_‐4S**. The steep slope between 0.6 and 0.8 equivalents indicates a morphological change from fibers to globular micelles. (d‐f) Cryo‐TEM measurements show fibers from pure **BTA** (1 mM) (d), a dense network of fibers from the **BTA** co‐assembly with 0.2 equivalents of **C_18_‐4S** (e), and short fibers and mainly hard visible small spherical micelles from the **BTA** co‐assembly with 1.0 equivalents of **C_18_‐4S** in PBS (f).

All surfactants have been investigated in different stochiometric ratios to the amount of **BTA** using UV‐vis spectroscopy (Figure S3). As our synthetic library includes surfactants with different headgroup architectures (i.e., 2‐, 4‐, and 8‐arm structures), we selected the 4‐arm dendritic surfactant **C_18_‐4S** as the lead candidate. This selection was motivated by its balanced amphiphilic character, which is expected to promote stable and well‐defined co‐assembly behavior. Due to their lack of an extended chromophore system, the surfactants absorb very weakly in 190–320 nm range. Thus, the absorbance of the co‐assemblies in Figure S3 is primarily due to **BTA** absorption. Nevertheless, morphological changes of the co‐assemblies induced by surfactants can be seen by changes in the absorption profiles. In our previous work, we investigated the structural transitions of **BTA**‐surfactant co‐assemblies from double helices to single fibers, shorter fibers, and finally spherical micelles [30]. A similar behavior was found here. With increasing ratio of surfactant, the co‐assembly morphology changes. However, this occurs at lower **BTA**‐surfactant ratios, indicating the anionic *SupraVir* surfactants’ higher disruptive strength in detail. Pristine **BTA** double helices (Figure 2b, blue) display characteristic double absorption peaks at 212 and 226 nm. For surfactant quantities below 0.6 equivalents, only minor changes are observed. We believe that double helices and an increasing number of single fibers are present here [29, 30]. Above 0.6 equivalents, an absorption shift towards 196 nm is observed (Figure 2c), indicating the presence of spherical micelles. Generally, those surfactants with more charges (4‐/8‐arm surfactants) exhibit the UV shift at lower equivalents ( > 0.6 eq) compared to the less charged 2‐arm surfactants (> 1.0 eq, Figure S4). However, the single‐arm variant sodium dodecyl sulfate (**SDS**) behaves differently. The absorption shift occurs above 0.6 equivalents (Figure S4), indicating a disruptive strength similar to that of **C_18_‐4S**.

To further elucidate the supramolecular aggregates, we examined different molar equivalents of **C_18_‐4S** using cryo‐TEM. All samples were prepared in PBS to simulate an in vitro test environment. In accordance with previous studies, pure **BTA** formed fibrous assemblies, as shown in Figure 2d [26]. The addition of a small amount of **C_18_‐4S** (0.2 equivalents) did not disrupt the **BTA** fibers, as shown in Figure 2e. Nevertheless, both micrographs reveal a **BTA** layer at the air‐water interface (Figure S5). As previously discussed, the so‐called fingerprint impacts the ratio of **BTA** and surfactant in solution. However, the surface‐to‐volume ratio of the cryo‐TEM grid is much larger compared to that of the quartz cuvette or 96‐well plate [30]. The addition of 1.0 molar equivalents of **C_18_‐4S** significantly disrupts the fiber morphology. This mixing resulted in the predominant appearance of hardly visible co‐micelles (Figure 2f) due to the solubilizing effect of the surfactant at this ratio. We attribute the disruption of **BTA** fibers by the surfactant to a similar mechanism previously proposed, whereby solubilization by surfactants prohibits the directional hydrogen bonding required for one‐dimensional fiber formation [30]. In this work, we mainly differentiate between fibers and micelles, since these morphologies impact antiviral activity. We propose that the micrometer‐long fibers can wrap the virus particles (100–200 nm) efficiently. Therefore, it is advantageous to keep the surfactant ratio low to ensure long supramolecular fibers doped with the *SupraVir* surfactant to bind the viruses. It is important to note that the transition between a double helix, a single fiber, and micelles has no clear borders, and we only take micrographs at certain ratios. We have experimentally demonstrated that the most significant structural control parameter is the surfactant ratio, with higher surfactant content causing a transition from long fibers to predominantly micellar aggregates. In the following, we will refer to a mixture with 0.2 equivalents of surfactant as a co‐assembly fiber and describe a sample with 1.0 equivalent of surfactant as predominantly micellar structure.

**FIGURE 3:**
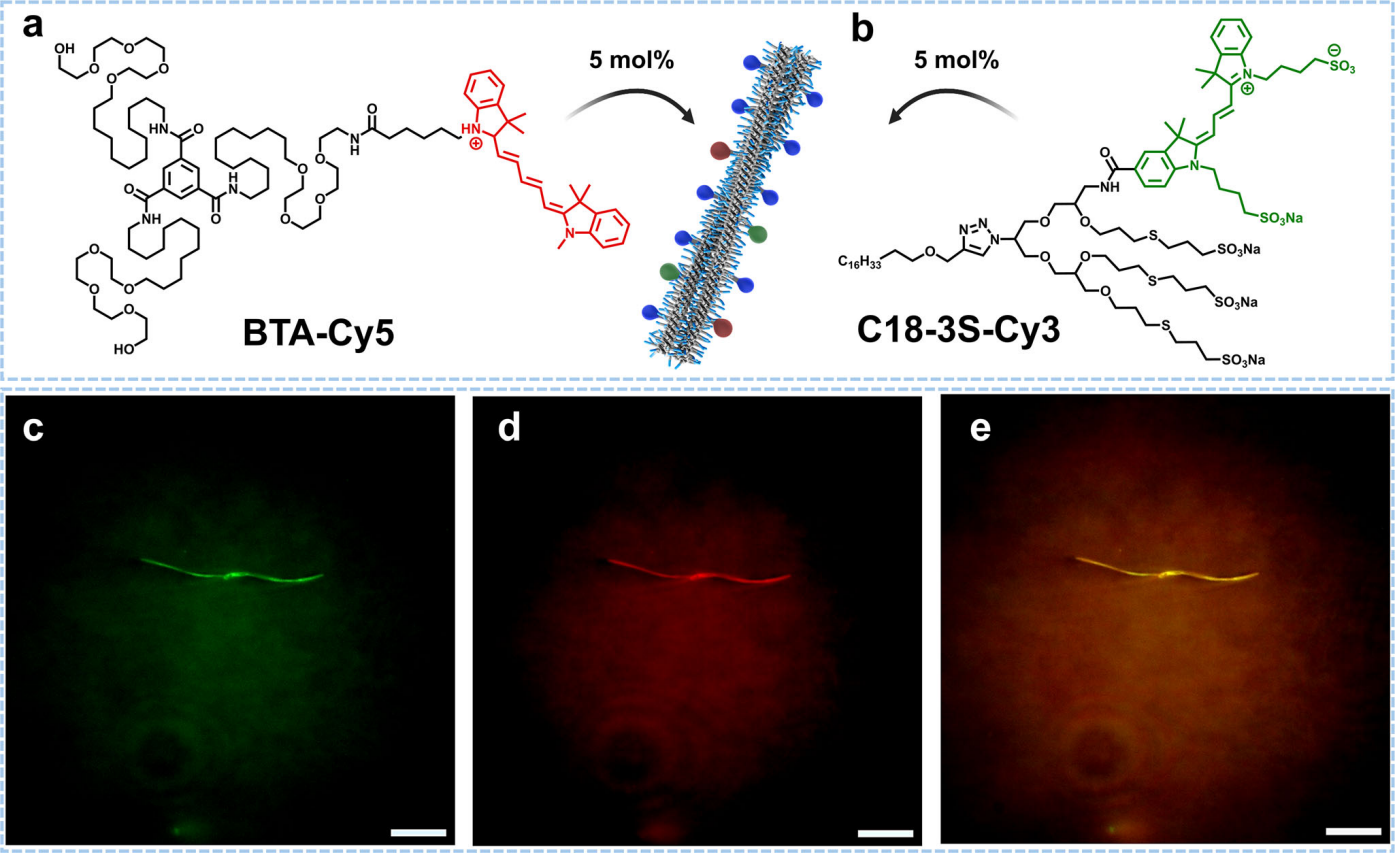
(a) Structure of Cy5‐labeled **BTA** [37]. (b) Structure of Cy3‐labled **C_18_‐4S** derivative (synthetic procedure in SI). Total internal reflection fluorescence (TIRF) microscopy of **BTA** co‐assembly with 0.2 equivalents **C_18_‐4S**. (c) Cy3 channel, (d) Cy5 channel, and (e) merged channel. The Pearsons correlation coefficient was calculated to 0.97, indicating an efficient colocalization of the dye‐labeled moieties. Sample of 50 µM of **BTA** (5 mol% **BTA**‐Cy5) and 0.2 molar equivalents of **C_18_‐4S** (5 mol% **C_18_‐3S‐Cy3**). Scale bar  =  10 µm.

To detect the intercalation of highly charged surfactants into the **BTA** scaffold, we performed colocalization experiments using total internal reflection fluorescence (TIRF) microscopy. We added fluorescently labeled monomers and surfactant (5 mol%) into the co‐assemblies using **BTA**‐Cy5 [39] and C_18_‐3S‐Cy3 (synthetic procedure in SI). Figure 3 shows the labeled monomers and the corresponding fluorescence channels for Cy3, Cy5, and the merged image. For **BTA** mixtures with 0.2 equivalents of **C_18_‐4S**, fibrous aggregates were observed that exhibited fluorescence in both channels, indicating co‐assembly of monomers and surfactants (Figure 3c–e). Given the 100–200 nm resolution limit of the TIRF microscope, the objects shown in Figure 3 and Figure S6, with micrometer length and sub micrometer thickness are most likely bundles of individual **BTA**‐surfactant fibers, possibly formed during the blotting process. TIRF microscopy was used to proof the colocalization of surfactant (Cy3) and **BTA** (Cy5) within the same fiber. The Pearsons correlation coefficient was calculated to be 0.97, indicating efficient colocalization of the dye labeled moieties. Consistent with the TEM images, fibrous structures were absent at higher **C_18_‐4S** ratios of (1.0 eq) due to surfactant‐induced disruption (Figure S7).

In conclusion, spectroscopic and microscopic data confirmed the initial incorporation of surfactants into **BTA** fibers and a morphological change (dissolution) of **BTA** fibers due to excessive ionic surfactant addition. Compared to our previous work with chiral non‐ionic surfactants, disruption of fiber morphology occurs at significantly lower surfactant equivalents in the current study [30]. To investigate HSV‐1 inhibition based on multivalency, we examined the system with surfactant equivalents ranging from 0.2, and 1.0 relative to the **BTA**. We then differentiate a fibrous morphology at 0.2 equivalents and a micellular morphology at 1.0 equivalents.

**FIGURE 4:**
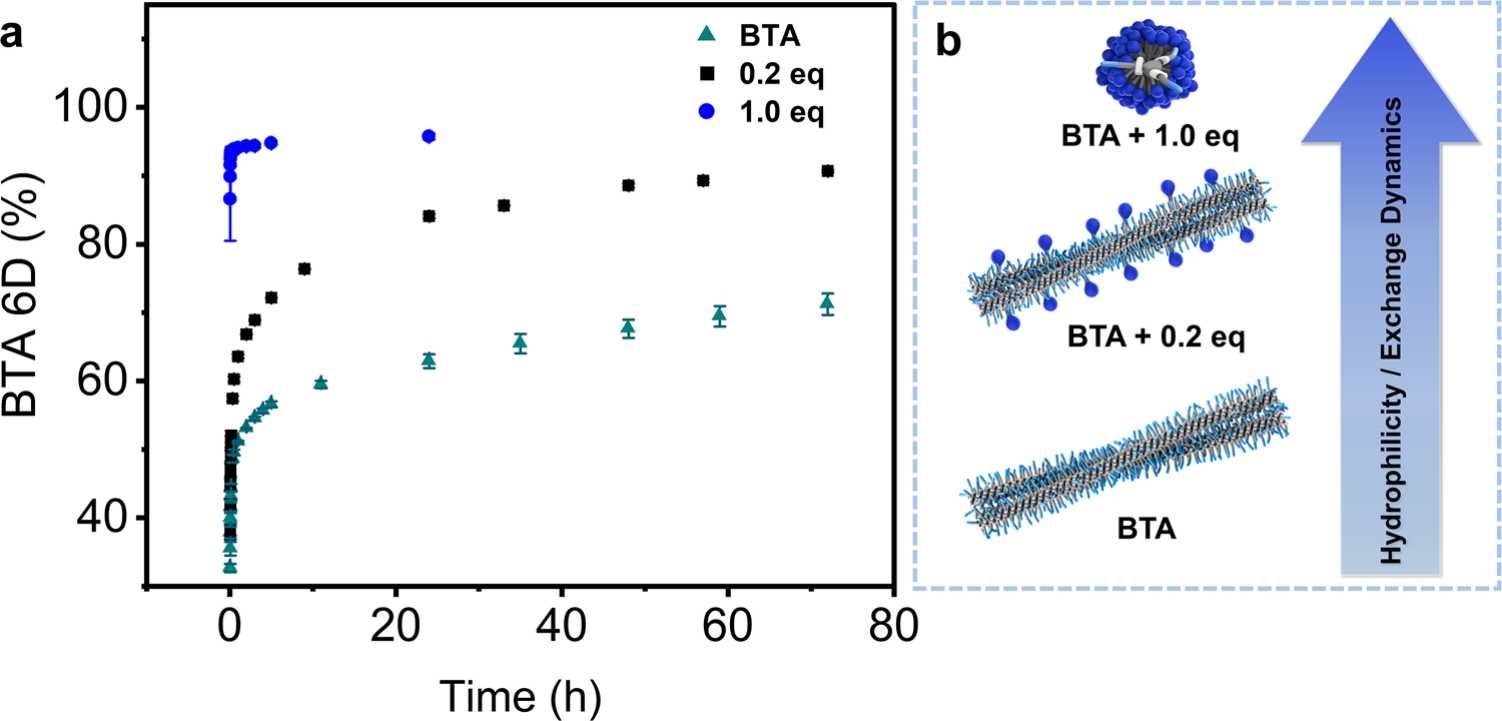
Hydrogen‐deuterium exchange experiments combined with mass spectrometry (HDX‐MS). (a) HDX‐MS results of **BTA** co‐assemblies with **C_18_‐4S** from a 5 µM **BTA** solution containing 0.2 or 1.0 molar equivalents of **C_18_‐4S,** compared to the previously studied exchange dynamics of pure **BTA** fiber. Ratio of **BTA**6D, in which all labile amide and hydroxy protons have been exchanged with deuterium, is plotted against the time at which the exchange occurred [38]. (b) Visualization of self‐assembled **BTA** aggregates and **BTA** co‐assemblies with **C_18_‐4S**, which increase in hydrophilicity and exchange dynamics.

Dynamics and flexibility of the co‐assembled fibers are necessary for better adaptation towards the virus morphology. We performed hydrogen‐deuterium exchange experiments coupled with mass spectrometry to elucidate these requirements [40, 41]. The time‐dependent exchange results of the labile amide and hydroxy protons of **BTA** are shown in Figure 4a. One **BTA** molecule has three hydroxyl hydrogen atoms at the peripheries of the tetra(ethylene glycol) and three amide hydrogen atoms in the core. When exposed to the surrounding D_2_O, the three OH groups immediately exchange to OD, resulting in **BTA**3D. The exchange rate of the NH groups, however, is much slower due to the reduced water accessibility in the hydrophobic pocket of the supramolecular fiber. Thus, **BTA**6D provides information about exchange dynamics and fiber stability. We observed a clear increase in the exchange of the inner amide protons with increasing ratios of the sulfonated surfactant **C_18_‐4S**. With 0.2 equivalents, the exchange behaviour is similar to that of pure **BTA**, especially for the initial exchange of up to 65%. The secondary exchange rate is steeper, reaching 80% **BTA**6D within 20 hours. In the subsequent 48 hours, the overall 6D rate increases to 85%, with a slope similar to that of pure **BTA**. Small surfactant ratios increase fiber dynamics by facilitating monomer exchange, which can be beneficial for morphological adaptation to virus shape. Full exchange of all labile protons within minutes after dilution of the 1:1 **BTA** sample corresponds to the expected dynamics of the observed small micellar co‐assemblies.

### 2.4 Cell‐Viability

The typical toxicity of small molecular amphiphiles limits their use in biomedicine. First, we investigated the cell viability of *SupraVir* surfactants separately from **BTA**. Cell viability assays using the CCK‐8 cell counting assay have been performed on Vero E6 cells up to 2 mM stock concentrations. Sigmoidal dose‐response fits were used to determine the 50% cytotoxic concentrations (CC_50_), thus are shown in Figure S9. **BTA** is known to be nontoxic and showed no toxicity against Vero E6 cells (Figure S8) [28]. All surfactants are less toxic than the commercially available **SDS** (0.30 ± 0.01 mM). In general, the surfactants exhibit CC_50_ above their CMCs. However, there is constant exchange of free surfactants between the micelle and its surroundings. This exchange contributes to cell‐toxicity through interaction between the free alkyl chain and the cell membrane [42]. Accordingly, *SupraVir* surfactants with longer alkyl chains (C_18_) showed increased toxicity compared to those with shorter chains (C_12_). For example, **C_12_‐2S** (1.90 ± 0.13 mM) has a higher CC_50_ compared to **C_18_‐2S** (0.77 ± 0.12 mM). This phenomenon is well reported in the literature [43, 44]. On the other hand, larger headgroups seem to reduce cell toxicity. For instance, the higher/more branched **C_18_‐4S** (1.80 ± 0.12 mM) has a higher CC_50_ than **C_18_‐2S**. Lastly, headgroup structure affects toxicity; the linear **C_18_‐lTG‐4S** (0.80 ± 0.06 mM) is more toxic than the dendritic **C_18_‐4S**.

Therefore, our amphiphilic molecular designs, which include dendritic headgroup structures and shorter alkyl chains, lead to better cell viability. However, it is crucial to strike the right balance between hydrophilic and hydrophobic components in order to achieve effective and stable intercalation into the **BTA** while presenting an effective number of functional groups for the targeted application. **C_18_‐4S** appears to be a good compromise, as it is much less toxic than the 2‐arm derivative.

**FIGURE 5:**
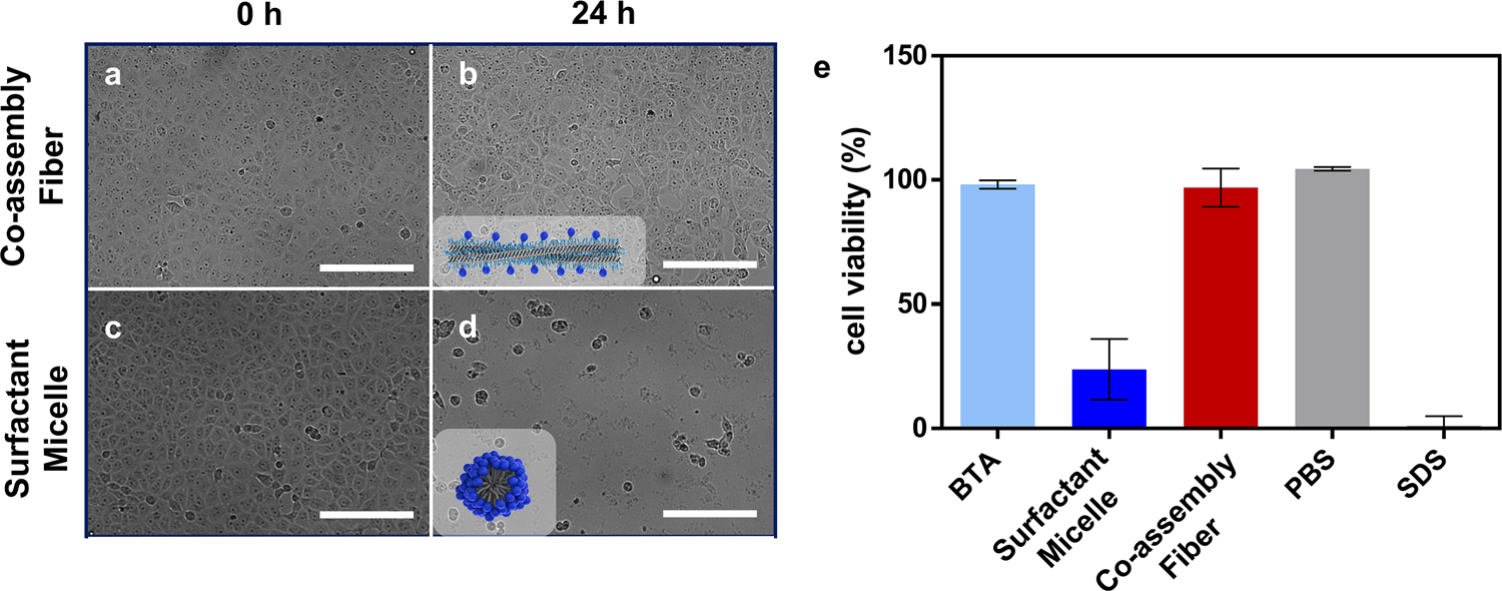
Investigation of cell viability of **BTA**, **C_18_‐4S** and a co‐assembly with 0.2 eq on Vero E6 cells. (a) Live cell imaging over 24 h visualizes cell toxicity through morphological changes of the cell. Disruption of the cell membrane leads to cell death. (a‐b) **BTA** + 0.2 eq **C_18_‐4S** at a surfactant concentration of 2 mM, shows no membrane disruption or toxicity. (c‐d) **C_18_‐4S** (2 mM) shows high cellular toxicity by disrupting cell membranes. Scale bars correspond to 200 µm. (e) CCK‐8 assay of the same samples is performed and compared to a 2 mM **BTA** solution, PBS, and **SDS** (1 mM).

We wanted to see how the supramolecular polymer scaffold affected cell viability. To do this, we used live‐cell imaging to study **C_18_‐4S** at 2 mM, which is higher than its CC_50_ (1.80 ± 0.12 mM). We compared this to a **BTA** co‐assembly mixture of 2 mM **C_18_‐4S** at a **BTA**–surfactant ratio of 5:1. When **BTA** was present (Figure 5a,b, Movie S1), no cell toxicity was visible. However, the pure **C_18_‐4S** was highly toxic to cells at that concentration (Figure 5c‐d, Movie S2). PBS and SDS were used as control experiments (Figure 5e). When the surfactant was used together with **BTA**, fibers could be seen in the cryo‐TEM and TIRF micrographs (Figures 2 and 3). These fibers show higher CC_50_ values. It is important to mention that co‐assemblies at **BTA** concentrations of 10 mM show high viscosity due to their hydrogel nature. To study this effect, we did a control experiment in which we used 2 w% methylcellulose as a hydrogel backbone. We added different surfactants to the hydrogel backbone. Pure methylcellulose was not toxic to cells. For the mixtures with surfactants, the same CC_50_ values could be obtained as for the pure surfactants (Figures S9 and S10). Based on this and previous investigations, we assume that the intercalation of the amphiphile alkyl chain into **BTA** nanofibers prevents them from interacting with cell membranes, thereby reducing the toxicity [29, 30]. The viscosity itself has no effect on cell viability.

### 2.5 HSV‐Inhibition

The low cell toxicity of the **BTA**‐*SupraVir* co‐assembly enabled us to advance to the pivotal phase of the virus inhibition assays. We performed pre‐infection inhibition assays to validate the effectiveness of different surfactants and co‐assemblies against HSV‐1, in which Vero E6 cells were preincubated with supramolecular (co‐)assemblies for 45 min, before HSV‐1 was added. The data obtained (Figure S11) revealed an unexpected result: pure **BTA** inhibited HSV‐1 infection with a half‐maximal inhibitory concentration (IC_50_) of 0.42 ± 0.02 mM. Due to the absence of functional groups that could interact with HSV‐1, this result could be explained by hydrogelation of the dense fiber network at this concentration.

The network could prevent virus‐cell interaction by size exclusion [29]. This was investigated by performing a control experiment in which **BTA** was preincubated with HSV‐1 for 45 min and then applied to the cells, which showed no inhibition. In this case, the virus‐sample mixture was homogenously applied to the cells. Nevertheless, this interesting observation complicates the interpretation of the inhibition of the functional co‐assemblies. Viscosity depends on the surfactant ratio as well as the total concentration. As the **BTA** concentration increases, so does the viscosity. However, the addition of surfactant breaks down the supramolecular fibers, thereby lowering the viscosity. Therefore, we proceeded with preincubating co‐assemblies and the virus before adding them to the cells to ensure that the detected inhibition is caused by surfactant‐virus interactions.

All synthesized *SupraVir* surfactants were tested individually against HSV‐1 and demonstrated inhibition in the micromolar range (Figure S12). Certain trends were observed among the different surfactants. For example, surfactants with longer alkyl chains showed lower IC_50_ values: **C_12_‐2S** (540 ± 29 µM) and **C_18_‐2S** (180 ± 11 µM). All C_18_ surfactants have lower IC_50_ values than commercial **SDS**. This can be explained by the stronger interaction of the longer chains with viral phospholipid membranes, similar to the higher cytotoxicity of C_18_ surfactants, as shown in Figure S9 and discussed in the previous section [45]. Secondly, headgroup size impacts inhibition in a nonlinear manner. The four‐arm surfactant **C_18_‐4S** (120 ± 27 µM) exhibited the lowest IC_50_ values compared to **C_18_‐2S** (180 ± 11 µM) and **C_18_‐8S** (260 ± 60 µM). **C_18_‐lTG‐4S** (140 ± 10 µM) has a linear structure and shows no observable difference compared to **C_18_‐4S**, however, a factor of greater than two was observed in terms of cell viability. The idea behind **C_18_‐lTG‐4S** was to investigate a surfactant derivative with the same charge density in its headgroup but with a different morphology. We hypothesized a different intercalation into the **BTA** stack and therefore a different activity. This was not the case; as it turned out that the antiviral effect was the same. Charge density seems to matter the most in terms of HSV‐1 inhibition. Overall, **C_18_‐4S** shows the best inhibition (IC_50_: 120 ± 27 µM), while cell toxicity has a CC_50_ value 15 times higher (CC_50_: 1.80 ± 0.12 mM). This indicates a potential therapeutic window, where antiviral activity is present without cytotoxicity. The balance between a C_18_ chain and a sulfonated, four‐arm surfactant appears favorable in terms of hydrophilicity and hydrophobicity. Thus **C_18_‐4S** is the best candidate for inhibition among pure surfactants while ensuring cell viability. However, the goal is to improve inhibition while maintaining good cell viability by co‐assembling with the supramolecular polymer **BTA**.

**FIGURE 6:**
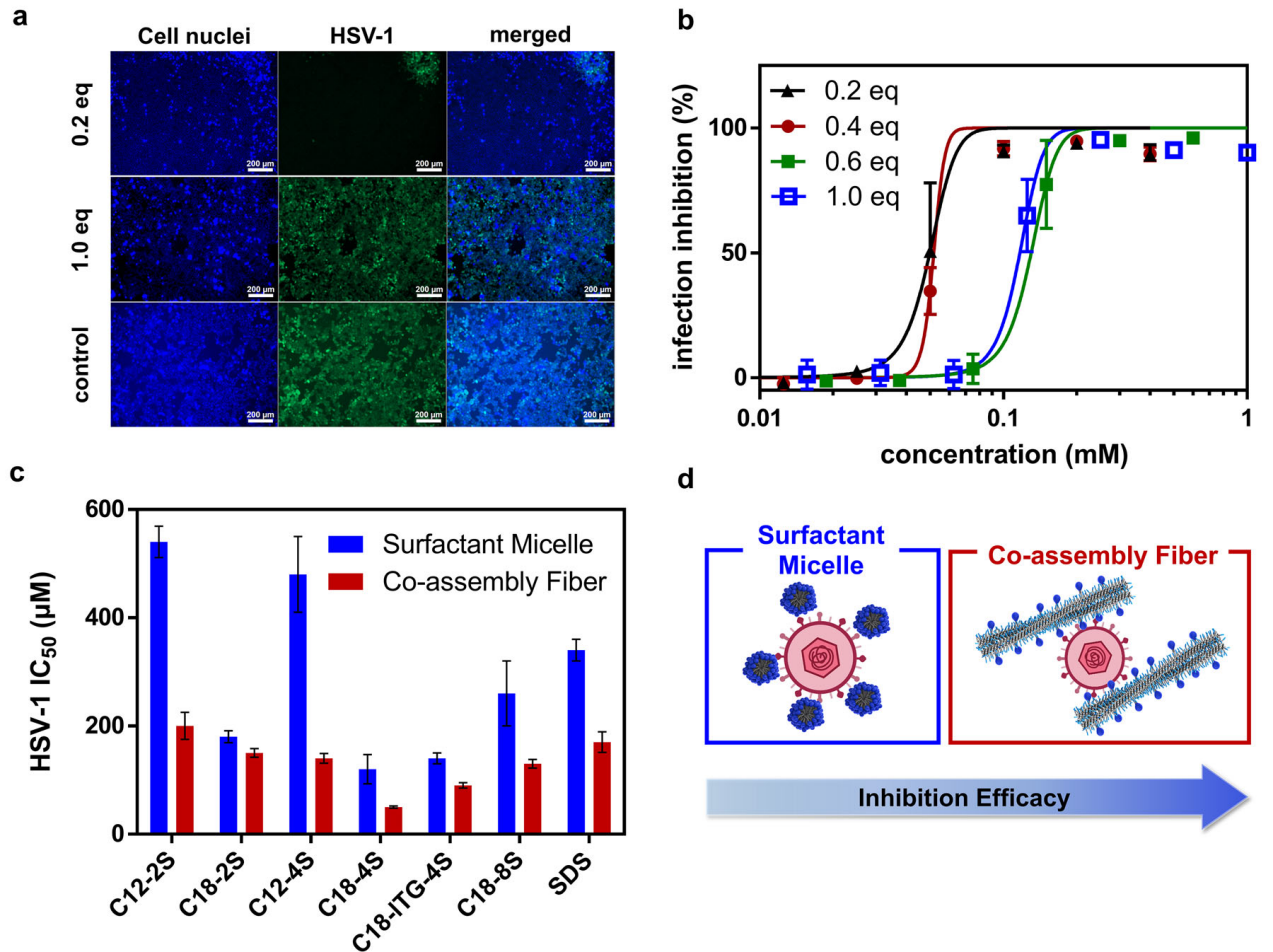
HSV‐1 inhibition of **BTA** co‐assemblies with different **C_18_‐4S** ratios. (a) Pre‐infection inhibition assay against HSV‐1. A comparison of optical microscope images using the DAPI channel (for cell nuclei) and the GFP channel (expressed by HSV‐1 infected Vero E6 cells) for **BTA** co‐assemblies with 0.2 and 1.0 eq of **C_18_‐4S** at a surfactant concentration of 50 µM. Scale bar equals 200 µm. (b) Infection curves for **BTA** co‐assemblies plotted against **C_18_‐4S** concentration, fitted with a sigmoidal curve. Lower IC_50_ values are visualized for 0.2 and 0.4 eq (fibrous morphology) compared to 0.6 and 1.0 eq (micellar assemblies). Values are expressed by mean ± SD, *n* = 3. (c) A comparison of **BTA** co‐assemblies of all *SupraVir* surfactants with and without **BTA**. The co‐assembly fiber (red) indicates, 0.2 eq of surfactant is present in the **BTA** fiber, the surfactant micelle (blue) contains only the surfactant. The IC_50_ values obtained from pre‐infection inhibition assays are present in µM ± SD (*n* = 3). (d) The expected morphology of different **BTA** surfactant co‐assemblies correlated with their infection inhibition efficacy. Co‐assemblies of **BTA** and *SupraVir* surfactants are more effective than pure surfactants.

The impact of supramolecular co‐assembly on IC_50_ values is evident. We observed distinct variations in inhibition for **C_18_‐4S** within **BTA** assemblies (Figure 6a,b) at different molar ratios. Co‐assemblies containing 0.2 or 0.4 equivalents of **C_18_‐4S** exhibited an IC_50_ value of 50 µM, however, those containing 0.6 or 1.0 equivalents showed higher IC_50_ values of 120 µM. These differences correlate with the morphology of the co‐assemblies (Figure 2) and their ability to inhibit HSV‐1 (Figure 6d). At lower surfactant ratios, co‐assemblies formed micrometer‐long, flexible, dynamic fibers that demonstrated superior inhibition compared to pure surfactant or co‐assemblies with higher surfactant ratios, in which smaller co‐micelles predominated. Importantly, the statistical incorporation of surfactants into the **BTA** fibers results in a defined spatial distribution of charged groups along the fiber. The resulting flexible and adaptive supramolecular architecture facilitates efficient multivalent interactions, in contrast to the more densely packed and structurally constrained micellar systems. Secondly, the incorporation of small amounts of surfactant enhances monomer exchange and optimizes the balance between stability and adaptability of **BTA** fibers, whereas higher surfactant ratios destabilize the assemblies too much and drive them toward less effective micellar structures. Consequently, we selected **BTA** co‐assemblies with 0.2 equivalents of each *SupraVir* surfactant for further study.

Mixing **BTA** with surfactants increases the antiviral activity of C_12_ surfactants more effectively than C_18_ surfactants (Figure 6c and Figure S13). This can be calculated as an activity factor (IC_50_ (surfactant)/IC_50_ (co‐assembly), as shown in Table S3 and demonstrated by the difference in IC_50_ values in Figure 6c (surfactant > co‐assembly). The highest values are measured for C_12_ surfactants (2.7–3.4). We hypothesize that there is better intercalation into the **BTA** fiber since the hydrophobic moiety of the **BTA** holds a C_12_ chain, enabling a better fit. Nevertheless, **C_18_‐4S** co‐assemblies remain the most effective HSV‐1 inhibitors (lowest IC_50_ values). It cannot be excluded that free surfactant outside the **BTA** fiber impacts cell toxicity and HSV‐1 inhibition since the charged system is dynamic (Figure 5a). Considering the inhibitory effect per charge (Table S3) shows that 4‐ and 8‐arm surfactants outperform the 1‐ and 2‐arm surfactants. This indicates a multivalent effect: the surfactant‐doped fibers with more negative charges bind better to viral receptor groups. The balance between hydrophilicity and hydrophobicity is of great importance and governs both the intercalation of the surfactant into the **BTA** fiber and the virus inhibition of the co‐assembly. **C_18_‐8S** is more hydrophilic than **C_18_‐4S**, therefore the intercalation into the **BTA** is less stable, leading to reduced virus inhibition.

## 3 Conclusion

In this study, we demonstrate how the supramolecular co‐assembly of **BTA** with simple anionic surfactants affects cell viability and virus binding. Using a two‐fold click chemistry approach, we synthesized a series of sulfonated surfactants with different headgroup sizes and alkyl chain lengths (*SupraVir* surfactants). These *SupraVir* surfactants form small micellar aggregates if pure or in equimolar ratios with **BTA**. However, they are incorporated into the fibrous morphology governed by **BTA** at low proportions (< 1). The effective intercalation of the surfactant's alkyl tail into the hydrophobic interior of **BTA** results in lower cytotoxicity and higher effectiveness against HSV‐1. This result highlights a morphological advantage of fibrous **BTA** co‐assemblies over micellar aggregates, which can be explained by two key differences. The fibrous co‐assemblies display their functional surfactants in micrometer‐long fibers that wrap the virus more efficiently than nanometer‐sized spherical micelles. Second, the exchange dynamics and thereby the stability of the aggregates play a major role. The fibers exhibit an ideal mixing regime at 0.2 equivalents of surfactant, at which they display improved dynamics in comparison to pure BTA, while maintaining enough stability for efficient viral inhibition.

Furthermore, all dendritic oligo‐glycerol surfactants, particularly the C_18_ surfactants, exhibit significantly lower toxicity than commercially available **SDS** while enhancing HSV‐1 inhibition. Co‐assemblies with C_12_ surfactants provide a great increase in antiviral activity (factors of 2.7‐3.4 compared to pure surfactants) throughout intercalation in **BTA**. Nevertheless, we believe that stronger interactions between **BTA** and surfactant molecules are necessary to achieve even lower IC_50_ values. To this end, it is important to prevent surfactants from being released from the supramolecular framework while maintaining dynamic co‐assembly.

In summary, we provide an easily accessible synthetic pathway for effective, virus‐inhibiting surfactants. We demonstrate that non‐covalent synthesis using different small monomeric building blocks that assemble into larger functional systems can be a promising approach for creating novel, potent, and dynamic bioactive supramolecular aggregates.

## Author Contributions

**Christian Zoister**: conceptualization, writing – original draft, writing – review and editing, investigation. **Boris Schade**: investigation, writing – review, and editing. **Paulina Sittinger**: investigation, writing – review, and editing. **Xianwen Lou**: investigation, writing – review, and editing. **Esmee de Korver**: investigation, writing – review, and editing. **Freek V. de Graaf**: writing – review and editing, investigation, supervision. **Ranen Etouki**: writing – review and editing, investigation. **Chuanxiong Nie**: writing – review and editing, investigation. **Kai Licha**: writing – review and editing, investigation. **Kai Ludwig**: writing – review and editing, visualization. **Abhishek Kumar Singh**: conceptualization, supervision, investigation, writing – review and editing. **E. W. Meijer**: conceptualization, funding acquisition, resources, writing – review and editing. **Rainer Haag**: conceptualization, funding acquisition, resources, writing – review and editing.

## Conflicts of Interest

The authors declare no conflicts of interest.

## Supporting information




**Supporting File 1**: anie73573‐sup‐0001‐SuppMat.docx.

**Supporting File 2**: anie73573‐sup‐0002‐MovieS1.mp4.

**Supporting File 3**: anie73573‐sup‐0003‐MovieS2.mp4.

## Data Availability

The data that support the findings of this study are available from the corresponding author upon reasonable request.
