# Supplementary material for: Supramolecular Polymer‐Surfactant Co‐Assemblies for Multivalent HSV‐1 Inhibition
Source: Angew Chem Int Ed Engl. 2026 Jul 26;65(39):e8702628. doi: 10.1002/anie.8702628 (PMC13592328; doi:10.1002/anie.8702628)
Supplement: Supplementary file 1 [file ANIE-65-e8702628-s002.docx]

Supporting Information for

Supramolecular Polymer-Surfactant Co-assemblies for Multivalent HSV-1 Inhibition

Christian Zoister,^[a,b]^ Boris Schade,^[c]^ Paulina Sittinger,^[a]^ Xianwen Lou,^[b]^ Esmee de Korver,^[b]^ Freek V. de Graaf,^[b]^ , Ranen Etouki,^[a]^ Chuanxiong Nie,^[a]^, Kai Licha, ^[a]^ Kai Ludwig,^[c]^ Abhishek Kumar Singh*^[a]^, E. W. Meijer,*^[b, d, e]^ and Rainer Haag*^[a]^

_________________________________________________________________________________

[a] Institute of Chemistry and Biochemistry, Freie Universität Berlin, Takustrasse 3, 14195 Berlin, Germany

[b] Institute for Complex Molecular Systems, Laboratory of Macromolecular and Organic Chemistry, Eindhoven University of Technology, PO Box 513, 5600 MB Eindhoven, The Netherlands

[c] Forschungszentrum für Elektronenmikroskopie, Core-Facility BioSupraMol, Institute of Chemistry and Biochemistry

Freie Universität Berlin, Fabeckstraße 36a, 14195 Berlin, Germany

[d] School of Chemistry and RNA Institute, University of New South Wales, Sydney, New South Wales 2052, Australia

[e] Max Planck Institute for Polymer Research, Ackermannweg 10, 55128 Mainz, Germany

* corresponding authors: abhikmc@zedat.fu-berlin.de, e.w.meijer@tue.nl, haag@zedat.fu-berlin.de

# Table of Content

[Table of Content	1](#_Toc216887120)

[Materials and Methods	2](#_Toc216887121)

[1.1	Synthesis of Individual Compounds	8](#_Toc216887122)

[1.2	Physicochemical Properties of Surfactants	39](#_Toc216887123)

[1.3	Dynamic Light Scattering (DLS)	40](#_Toc216887124)

[1.4	Critical micelle concentration	41](#_Toc216887125)

[1.5	Co-assembly Investigations	42](#_Toc216887126)

[1.6	Cryo-TEM	44](#_Toc216887127)

[1.7	TIRF microscopy	45](#_Toc216887128)

[1.8	Cell viability	46](#_Toc216887129)

[1.9	Pre-Infection Inhibition of HSV-1	48](#_Toc216887130)

[2.	Literature	52](#_Toc216887131)

[3.	Appendix	53](#_Toc216887132)

[3.1	NMR	53](#_Toc216887133)

## Materials and Methods

**Reagents**

All solvents were used in HPLC quality, as well as deuterated solvents. All reagents and solvents were used as purchased from commercial suppliers, without any prior purification. All reactions which involved air- or water-sensitive compounds were carried out in a dried flask under an argon atmosphere.

**Thin layer chromatography**

Thin layer chromatography (TLC) analysis was performed on Merck silica gel 60, Fluorescenceindikator F_254_. A solution of KMnO_4_ was used as a staining solution. The solution contains 1.5 g of KMnO_4_, 10 g K_2_CO_3_, and 1.25 mL 10% NaOH in 200 mL water.

**Column chromatography**

The preparative purification of mixtures by column chromatography was conducted on Merck silica gel 60 (0.040–0.063 mm). The different eluents used are described in the synthesis section.

**Dialysis**

All dialysis purifications were performed using Biotech cellulose ester (CE) dialysis tubing 100‑500 D, 31 mm. The solvents, PBS and later Milli-Q water were changed three times per day.

**NMR spectroscopy**

^1^H and ^13^C NMR spectra were recorded on Bruker Ultrashield 400 MHz spectrometers (400 MHz for ^1^H NMR; 100 MHz for ^13^C NMR) or JEOL Spectrometer ECZ600 (600 MHz for ^1^H NMR, 151 MHz for ^13^C NMR) or JEOL Spectrometer ECP500 (500 MHz for ^1^H NMR, 151 MHz for ^13^C NMR). Chemical shifts δ are given in ppm relative to TMS as an internal standard or relative to the resonance of the solvent (^1^H NMR: Chloroform: δ = 7.26 ppm; D_2_O: δ = 4.79 ppm; MeOD δ = 3.31 ppm; ^13^C NMR: Chloroform: δ = 77.00 ppm; MeOD: δ = 49.00 ppm). Deuterated solvents used are indicated in each case. Peak multiplicities are abbreviated as s: singlet; d: doublet; m: multiplet; t: triplet.

**Sample preparation**

All BTA and surfactant stock solutions were prepared by weighing a certain amount of the dry material in a glass vial using a microgram balance. The desired amount of PBS was added to the sample. The solution was vortexed, sonicated, heated up to 80°C for 15 minutes and stirred overnight at room temperature. The samples containing BTA and surfactant were prepared from these stock solutions and treated with the same protocol.

**Ultraviolet absorption spectroscopy (UV)**

UV absorption spectra were measured on a JASCO J-815 CD spectrometer. The measurements were performed in Quartz cuvettes (Hellma) with a pathlength of 1 mm (250 µM). Standard sensitivity, a D.I.T of 2 seconds, a bandwidth of 1.0 nm, a data pitch of 2 nm and a scanning speed of 50 nm/min were used. The spectra were recorded from 350 nm to 190 nm. The spectra were corrected with a baseline of the corresponding solvent that was recorded before the measurement.

**DLS**

Dynamic light scattering (DLS) measurements of size and size distribution were measured using a Malvern Zetasizer Ultra (Malvern Instruments Limited, U.K.) at a constant temperature of 25 °C in 150 mM PBS buffer solution at a fixed concentration of 1 mM. The samples were prepared as described above and calibrated for 2 min before the measurement was performed.

**Zeta-Potential**

ζ-Potential measurements were performed using a Malvern Zetasizer Ultra (Malvern Instruments Limited, U.K.) at a constant temperature of 25 °C in 15 mM PBS buffer solution at a fixed concentration of 1 mM. The samples were prepared as described above and calibrated for 2 min before the measurement was performed.

**Cryo-TEM**

Perforated carbon film covered microscopical 200 mesh grids (R1/4 batch of Quantifoil, MicroTools GmbH, Jena, Germany) were cleaned with chloroform and hydrophilised by 60 s glow discharging at 10 μA in a EMSCOPE SC500. 4 μl aliquots of the amphiphile solution were applied to the grids before automatic blotting and plunge freezing in a FEI Vitrobot Mark IV (Thermo Fisher Scientific Inc., Waltham, Massachusetts, USA) was performed, using liquid ethane as cryogen. For measurements, the vitrified grids were transferred to the autoloader of a FEI TALOS ARCTICA™ electron microscope (Thermo Fisher Scientific Inc., Waltham, Massachusetts, USA). The microscope is equipped with a high-brightness field-emission gun (XFEG) operated at an acceleration voltage of 200 kV. Micrographs were acquired on a FEI Falcon 3 direct electron detector (Thermo Fisher Scientific Inc., Waltham, Massachusetts, USA) at a nominal magnification of 28,000, corresponding to a calibrated pixel size of 3.75 Å/pixel.

**Fluorescence microscopy**

TIRF images were acquired with a Nikon N-STORM microscopy system. Fluorescence was collected using a Nikon×100, 1.4NA oil immersion objective and passed through a quad-band pass dichroic filter (97335 Nikon). Cy3 was excited using a 561 nm laser while Cy5 was excited using a 647 nm laser. Images were recorded with an EMCCD camera (ixon3, Andor, pixel size 0.17 μm). The samples were prepared from a 0.25 mM stock by diluting with 2 mM NaCl solution to the desired concentration. Samples were imaged in a μ-Slide 8 Well plate with No. 1.5 glass coverslip bottom suitable for microscopy.

**Hydrogen Deuterium Exchange Mass Spectrometry (HDX-MS)**

Hydrogen deuterium exchange mass spectrometry (HDX-MS) experiments were carried out using a Xevo^TM^ G2 QTof mass spectrometer (Waters) with a capillary voltage of 2.7 kV, a sampling cone voltage of 20 V and extraction cone voltage of 4.0 V. The source temperature was set at 100 °C, the desolvation temperature at 400 °C, the cone gas flow at 10 L h^-1^ and the desolvation gas flow at 100 L h^-1^. The sample solutions subjected to HDX were introduced into the mass spectrometer using a Harvard syringe pump (11 Plus, Harvard Apparatus) at a flow rate of 50 µL min^-1^. Samples of **nBTA** and surfactants were prepared at 0.5 mM of **nBTA** and diluted 100 times with D_2_O (including 0.5 mM sodium acetate to facilitate detection), resulting in a final concentration of 5 µM. The samples were stored at room temperature during the experiment. The H/D exchange was followed over time and the percentages of the different deuterated isotopes were calculated. The distribution of doubly charged ions ([BTA+2Na]^2+^) were used for the calculations, and the theoretical isotopic distributions were calculated with an IsoPro Software.

All **nBTA** species observed have isotopes that overlap with more deuterated analogues. For example, **nBTA** 3D has its first isotope with the same mass as monoisotopic mass of **nBTA** 4D, its second isotope with the same mass as the monoisotopic mass of **nBTA** 5D and its third isotope with the same mass as the monoisotopic mass of **nBTA** 6D.

Table S1: Theoretical isotope distribution of the doubly charged nBTA as calculated with the IsoPro Software, including a graphical representation. This would be the isotope distribution without the presence of D_2_O.

| **[BTA+2Na]^2+^** | |  |
| --- | --- | --- |
| m/z | Relative  abundance |  |
| 666.96 | 1 |  |
| 667.46 | 0.80 |  |
| 667.96 | 0.36 |  |
| 668.47 | 0.11 |  |
| 668.97 | 0.03 |  |
| 669.47 | 0.01 |  |

Although **nBTA** 3D and 6D are the most abundant species in the case of **nBTA** in D_2_O, the isotopes of 1D, 2D, 4D and 5D were also taken into account for the calculation. With the distribution values given in Table S1, we could calculate the intensities of the isotopes and subtract these from the measured intensities at each m/z value. For example, to get the intensity of **nBTA** 3D without the influence of isotopes, we would have to subtract the first isotope of **nBTA** 2D and the second isotope of **nBTA** 1D from the measured intensity at the monoisotopic m/z of **nBTA** 3D. We assume that the deuterated **nBTA**s have the same isotope distribution as the non-deuterated **nBTA**. The corrected intensity (I_nBTAnD_c_) of the monoisotopic peaks without the influence of the isotopes can be calculated with the constants in the table above and the following set of equations:

$$\text{I}_{\text{nBTA1D\_c}}\text{ =}\text{ I}_{\text{667.46}}$$

$$\text{I}_{\text{nBTA2D\_c}}\text{ = }\text{I}_{\text{667.96}}\text{ - 0.80I}{}_{\text{nBTA1D\_c}}$$

$$\text{I}{}_{\text{nBTA3D\_c}}\text{ =}\text{ I}_{\text{668.47}}\text{ - 0.80}\text{I}_{\text{nBTA2D\_c}}\text{ - 0.36}\text{I}_{\text{nBTA1D\_c}}$$

$$\text{I}_{\text{nBTA4D\_c}}\text{ = }\text{I}_{\text{668.97}}\text{ - 0.80}\text{I}_{\text{nBTA3D\_c}}\text{ - 0.36}\text{I}_{\text{nBTA2D\_c}}\text{ - 0.11}\text{I}_{\text{nBTA1D\_c}}$$

$$\text{I}_{\text{nBTA5D\_c}}\text{ = }\text{I}_{\text{669.47}}\text{ - 0.80}\text{I}_{\text{nBTA4D\_c }}\text{- 0.36}\text{I}_{\text{nBTA3D\_c }}\text{- 0.11}\text{I}_{\text{nBTA2D\_c}}\text{ - 0.03}\text{I}_{\text{nBTA1D\_c}}$$

$$\text{I}_{\text{nBTA6D\_c }}\text{= }\text{I}_{\text{669.97}}\text{ - 0.80}\text{I}_{\text{nBTA5D\_c}}\text{ - 0.36}\text{I}_{\text{nBTA4D\_c}}\text{ - 0.11}\text{I}_{\text{nBTA3D\_c}}\text{ - 0.03}\text{I}_{\text{nBTA2D\_c }}\text{- 0.01}\text{I}_{\text{nBTA1D\_c}}$$

With I_667.46_, I_667.96_, I_668.47_, I_668.97_, I_669.47_ and I_669.97_ the intensity at m/z = 667.46, 667.96, 668.47, 668.97, 669.47 and 669.97, respectively.

**Cell Viability (Vero E6)**

The cell viability of the BTA and co-assemblies were investigated on Vero E6 (ATCC number CRC-1586). The cells were cultured in Dulbecco’s Modified Eagle’s Medium (DMEM) supplemented with 10% FBS, 1% penicillin and streptomycin (all from Gibco BRL, Eggenstein, Germany). The cells were subcultured twice a week when they reached 90% confluency. For the cell viability assay, 90 µL of a cell suspension in DMEM containing 1×10^4^ cells/mL were seeded in each inner well of a 96-well plate and incubated for 3 days at 37 °C and 5% CO_2_. In the outer wells, were prepared with 90 µL DMEM without cells. After reaching confluency, 10 µL per concentration of the desired compound was added to the cells in duplicates and in addition to one outer well for the background correction. SDS (1%) and PBS served as controls. The cells together with the compounds were incubated for 48 h at 37 °C and 5% CO_2_. Afterwards the CCK-8 solution was added (10 µL/well), and the absorbance at a measurement wavelength of 450 nm and a reference wavelength of 650 nm was measured after 3 h incubation using a Tecan plate reader. Measurements were performed in duplicates and repeated three times. The cell viability was calculated by normalizing to the non-treated control after correction by the background using the Excel software. The graphs were plotted in GraphPad Prim 6.

**Live cell imaging**

Vero E6 cells were seeded in a 96 well plate and cultured till they achieved 90% confluency for the experiment. The different samples: 4S, 0.2 eq, 1.0 eq and BTA samples, each in 3 concentrations (above, at and below the CC-50 of C_18_-4S) were added to the cells. Therefore, 20 µl of medium was removed and then 20 µl of sample was added. The focus was set manually, and over a period of 24 h with an interval of 10 min brightfield images were taken with ZEISS Axio Observer 7 (Apotome. 2) within an incubator of 37 ^o^C and 5% CO_2_. Afterwards, the cells are measured by CCK‑8 with an incubation time of 3 h as described above.

**HSV Pre-Infection Inhibition**

HSV-1 with GFP modification was kindly provided by Dr. Jakob Trimpert and Prof. Klaus Osterrieder (Institut für Virologie, Freie Universität Berlin). For the pre-infection inhibition assay, 90 µL of a Vero E6 cell suspension in DMEM containing 1×10^4^ cells/mL were seeded in a 96-well plate and incubated for 3 days at 37 °C and 5% CO_2_. After removal of the medium and washing with PBS, 160 µL of medium and 40 µL of the first sample were added to the first well. A 2-fold dilution series was performed. The cells were preincubated with the samples for 45 min at 37 °C and 5% CO_2_. 50 µL of the diluted recombinant HSV-1‑GFP (multiplicity of infection: 0.1) was added. For the negative control 50 µL medium were added. The cells were incubated for 48 h at 37 °C and 5% CO_2_. In another experimental set-up (preincubation of BTA and virus) the virus and the sample were preincubated for 45 min before addition to the cells. After incubation, the cell nuclei were stained using Hoechst. The cells were fixed by 4% formaldehyde (>30 min). Afterwards, the cells were imaged by a fluorescent microscope (ZEISS Axio Observer 7 (Apotome.2)). The IC-50 was calculated by infection ratios of infected cells and all cells using the Image J plug-in from Kerkhoff et. al and GraphPad Prim 6 involving a dose-response-curve fit (four parameters).^[1]^

## 1.1 Synthesis of Individual Compounds

Scheme S1: General synthesis scheme for modular surfactants. Alkyl chain modification via ether synthesis introducing the alkyne. Monomer synthesis using the example of C18-4S. Starting from protected oligo-glycerol unit, the hydroxy group got transformed into an azide throughout mesylation. This was followed by allylation, leading to the G1-allyl as the monomer for double click chemistry. Cu(I)-catalyzed azide–alkyne cycloaddition (CuAAC) was used for the alkyl chain introduction. Finally, thiol-ene click chemistry enabled the introduction of sulfonates. This general synthesis route was modified by changing equivalents for the different surfactants. Some reactions have been performed and products used in our cationic amphiphile project.^[2]^

#### C_12_-alkyne

NaH (2.0 eq, 2.15 g, 53.7 mmol) was dissolved under dry conditions and ice cooling in THF (100 mL). First dodecanol (1.0 eq, 5.00 g, 26.8 mmol) and then propargyl bromide (1.2 eq, 3.38 g, 32.2 mmol) were added slowly to the reaction mixture. The reaction was carried out overnight at 50 °C. The progress of the reaction was monitored using thin layer chromatography (pure cyclohexane). Once the reaction was completed it was quenched using *iso*-propanol under ice cooling. The solvents were removed under reduced pressure and the residue was extracted using DCM/H_2_O (3 x 100 mL). The combined organic layers were dried over NaSO_4_ and purified by column chromatography using cyclohexane/EtOAc. The product (5.40 g, 92%) was obtained as a colorless oil.

**^1^H NMR** (600 MHz, CDCl_3_, ppm) δ = 4.13 (d, *J* = 2.4 Hz, 2H), 3.50 (t, *J* = 6.7 Hz, 2H), 2.41 (t, *J* = 2.4 Hz, 1H), 1.61 – 1.55 (m, 2H), 1.35 – 1.22 (s, 18H), 0.88 (t, *J* = 7.0 Hz, 3H).

**^13^C NMR** (151 MHz, CDCl_3_, ppm) δ = 80.0, 73.9, 70.3, 57.9, 31.9, 29.6, 29.6, 29.5, 29.4, 29.4, 29.3, 26.0, 22.6, 14.0.

**HRMS (ESI):** *m*/*z* calcd for C_15_H_28_NaO: 247.2040 [*M+*Na]^+^; found: 247.2038.

#### C18-alkyne

NaH (2.0 eq, 890 mg, 37.0 mmol) was dissolved under dry conditions and ice cooling in THF (50 mL). First octadecanol (1.0 eq, 5.00 g, 18.5 mmol) and then propargyl bromide (1.2 eq, 1.30 g, 22.2 mmol) were added slowly to the reaction mixture. The reaction was carried out overnight at 50 °C. The progress of the reaction was monitored using thin layer chromatography (cyclohexane). Once the reaction was completed it was quenched using *iso*-propanol under ice cooling. The solvents were removed under reduced pressure. The residue was extracted with DCM/H_2_O (3 x 100 mL). The combined organic layers were dried over NaSO_4_ and purified by column chromatography using cyclohexane/EtOAc. The product (5.30 g, 92%) was obtained as a colorless oil.

**^1^H NMR** (700 MHz, CDCl_3_, ppm) δ = 4.13 (d, *J* = 2.4 Hz, 2H), 3.51 (t, *J* = 6.7 Hz, 2H), 2.41 (t, *J* = 2.4 Hz, 1H), 1.60 – 1.58 (m, 6.6 Hz, 2H), 1.29 – 1.24 (m, 30H), 0.87 (d, *J* = 7.2 Hz, 3H).

**^13^C NMR** (176 MHz, CDCl_3_, ppm) δ = 80.2, 77.3, 77.2, 77.0, 74.1, 70.5, 58.2, 32.1, 29.9, 29.8, 29.8, 29.7, 29.7, 29.6, 29.5, 26.2, 22.8, 14.3.

**HRMS (ESI):** *m*/*z* calcd for C_21_H_40_NaO: 331.2977 [*M+*Na]^+^; found: 331.3115.

#### G0-allyl

Allylglycidylether (1.0 eq, 2.00 g, 17.5 mmol) and allylalcohol (2.0 eq, 2.04 g, 35.0 mmol) were dissolved in H_2_O and toluol (each, 40.0 mL). Afterwards potassium hydroxide (2.0 eq, 1.97 g, 35.0 mmol) and TBAB (0.1 eq, 0.56 g, 1.75 mmol) were added to the reaction mixture. The reaction mixture was stirred overnight at 50 °C. The crude product was extracted using EtOAc/H_2_O. Followed by further purification using distillation. G0-OH (2.20 g, 75%) was obtained as a colorless liquid.

**^1^H NMR** (600 MHz, CDCl_3_, ppm) δ 5.94 – 5.79 (m, 2H), 5.33 – 5.07 (m, 4H), 4.04 – 3.89 (m, 5H), 3.58 – 3.33 (m, 4H).

**^13^C NMR** (151 MHz, CDCl_3_, ppm) δ 134.8, 134.6, 134.4, 118.3, 117.8, 117.5, 117.4, 117.3, 117.2, 116.8, 116.3, 72.6, 72.4, 72.2, 71.9, 71.7, 71.6, 71.5, 71.4, 71.2, 71.1, 70.9, 70.6, 69.6, 69.3, 54.0, 29.2, 20.9, 14.2.

**HRMS (ESI):** *m*/*z* calcd for C_9_H_16_NaO_3_: 195.0997 [*M+*Na]^+^; found: 195.0996.

#### G0-N_3_

PPh_3_ (1.5 eq, 2.28 g, 8.71 mmol) was placed into a 100 mL flask. DIAD (1.5 eq, 1.76 g, 8.71 mmol, was slowly added under ice cooling. THF (20 mL) was added once the solid complex was formed. When the complex had dissolved, the ice bath was removed. Afterwards, the reaction mixture was slowly warmed to room temperature. G0‑OH (1.0 eq, 1.00 g, 5.81 mmol) was dissolved in 10 mL THF and carefully added to the mixture. In the next step, DPPA (1.5 eq, 2.40 g, 8.71 mmol) was added. Once two hours have passed the reaction was monitored via TLC. Once the reaction was completed, THF was removed, and the residue was extracted with EA/H_2_O (3 x 50 mL). The organic phases were combined and dried over Na_2_SO_4_. Then, the substance was purified by column chromatography (cyclohexane/EtOAc, 10:1). The product (0.36 g, 31%) was obtained as a colorless oil.

**^1^H NMR** (600 MHz, CDCl_3_, ppm) δ = 7.28 – 7.18 (m, 2H), 6.64 – 6.51 (m, 4H), 5.37 – 5.33 (m, 4H), 5.06 (tt, *J* = 9.7, 3.1 Hz, 1H), 4.95 – 4.88 (m, 2H), 4.88 – 4.84 (m, 2H).

**^13^C NMR** (151 MHz, CDCl_3_, ppm) δ = 134.3, 117.4, 72.4, 69.8, 60.8 ppm.

**HRMS (ESI):** *m*/*z* calcd for C_9_H_15_N_3_NaO_2_: 220.1062 [*M+*Na]^+^; found: 220.1064.

#### C_12_-2allyl

G0‑N3 (1.0 eq, 200 mg, 1.01 mmol) and C_12_‑alkyne (1.2 eq, 273 mg, 1.22 mmol) were placed into a 100 mL flask, dissolved in DMF (50 mL) and stirred well. Argon was bubbled through the solution for about 20 min. Afterwards, Copper acetate (0.40 eq, 55.8 mg, 0.41 mmol) was added to the reaction mixture. The reaction was heated up to 50 °C and stirred overnight. The reaction of the educts as well as the formation of the product was monitored using TLC. Once the reaction was complete, the solvents were removed under reduced pressure. Afterwards, the residue was extracted using DCM/H_2_O (3 x 50 mL) and washed with a saturated solution of EDTA. The organic phases were combined and dried over Na_2_SO_4._ Column chromatography was used for purification using hexane/EtOAc (10:1). The product (290 mg, 70%) was obtained as a colorless oil.

**^1^H NMR** (600 MHz, CDCl_3_, ppm) δ = 7.71 (s, 1H), 5.85 – 5.79 (m, 2H), 5.27 – 5.13 (m, 4H), 4.90 (p, *J* = 5.7 Hz, 1H), 4.62 (s, 2H), 4.00 – 3.93 (m, 4H), 3.86 (dd, *J* = 5.7, 1.7 Hz, 4H), 3.50 (t, *J* = 6.7 Hz, 2H), 1.58 (p, *J* = 6.9 Hz, 2H), 1.36 – 1.20 (m, 18H), 0.87 (t, *J* = 6.9 Hz, 3H).

**^13^C NMR** (151 MHz, CDCl_3_, ppm) δ = 145.40, 134.31, 123.02, 117.93, 72.64, 71.19, 69.09, 64.72, 61.02, 32.26, 30.24 – 29.49 (m), 26.48, 23.03, 14.46.

**HRMS (ESI):** *m*/*z* calcd for C_24_H_43_N_3_NaO_3_: 444.3202 [*M+*Na]^+^; found: 444.3242.

#### C_18_-2allyl

G0‑N3 (1.0 eq, 100 mg, 0.510 mmol) and C_18_‑alkyne (1.2 eq, 188 mg, 0.61 mmol) were placed into a 100 mL flask, dissolved in DMF (50 mL) and stirred well. Argon was bubbled through the solution for about 20 min. Afterwards, Copper acetate (0.40 eq, 27.9 mg, 0.20 mmol) was added to the reaction mixture. The reaction was heated up to 50 °C and stirred overnight. The reaction of the educts as well as the formation of the product was monitored using TLC. Once the reaction was complete, the solvents were removed under reduced pressure. The product was extracted using DCM/H_2_O (3 x 50 mL) and washed with a saturated solution of EDTA. The organic phases were combined and dried over Na_2_SO_4._ Column chromatography was used for purification using hexane/EtOAc (10:1). The product (97.4 mg, 38%) was obtained as a colorless oil.

**^1^H NMR** (700 MHz, CDCl_3_, ppm) δ = 8.04 (s, 1H), 5.51 – 5.42 (m, 2H), 4.94 – 4.79 (m, 4H), 4.29 (s, 2H), 4.01 – 4.00 (m, 1H), 3.70 – 3.61 (m, 4H), 3.61 – 3.57 (m, 2H), 3.56 – 3.52 (m, 2H), 3.32 – 3.28 (m, 2H), 1.44 (pd, *J* = 6.8, 2.5 Hz, 2H), 1.10 – 1.08 (m, 2H) 1.08 – 0.92 (m, 28H), 0.55 – 0.52 (m, 3H).

**^13^C NMR** (176 MHz, CDCl_3_, ppm) δ = 133.35, 116.99, 71.78, 68.03, 47.95, 29.17.

**HRMS (ESI):** *m*/*z* calcd for C_30_H_55_N_3_NaO_3_: 528.4141 [*M+*Na]^+^; found: 528.8859.

#### C_12_-2S

C_12_‑2allyl (1.0 eq, 161.3 mg, 0.380 mmol) was placed into a 50 mL reaction flask. MeOH (50 mL) was used as solvent. Sodium‑3‑mercaptopropyl sulfonate (4.0 eq, 297 mg, 1.53 mmol) and the photocatalyst 2,2‑dimethoxy‑1,2‑diphenylethan‑1‑one (0.40 eq, 39.2 mg, 0.150 mmol) were added to the reaction mixture. Argon was bubbled through the solution for about 20 min. The reaction was conducted under UV light (365 nm) for a duration of 6 hours. The reaction was monitored using TLC. Once the reaction was completed the solvent was removed under reduced pressure. The solid left in the flask was dissolved in water and dialyzed (100‑500 D) against water for 2 days. The water was changed three times a day. The residue was lyophilized. The product (71.1 mg, 23%) was obtained as a fluffy white solid.

**^1^H NMR** (700 MHz, MeOD, ppm) δ = 8.04 (s, 1H), 4.59 (s, 1H), 3.90 – 3.89 (m, 1H), 3.87 – 3.86 (m, 1H), 3.58 – 3.50 (m, 4H), 2.93 – 2.91 (m, 6H), 2.66 – 2.60 (m, 8H), 2.51 – 2.49 (m, 2H), 2.20 – 2.18 (s, 2H), 2.07 – 2.02 (m, 8H), 1.79 – 1.76 (m, 2H), 1.34 – 1.25 (s, 18H), 0.90 (t, *J*=7.1, 3H).

**^13^C NMR** (176 MHz, MeOD, ppm) δ = δ 70.73, 64.62, 51.43, 50.99, 38.20, 33.08, 31.66, 30.61 (t, *J* = 24.3 Hz), 29.04, 27.24, 26.18, 25.75, 24.04, 23.73, 14.44.

**HRMS (ESI):** *m*/*z* calcd for C_30_H_57_N_3_NaO_9_S_4_: 754.2875 [*M*]^–^; found: 754.2871.

#### C_18_-2S

C_18_‑2allyl (1.0 eq, 97.0 mg, 192 mmol) was placed into a 50 mL reaction flask. MeOH (50 mL) was used as solvent. Sodium‑3‑mercaptopropyl sulfonate (4.0 eq, 149 mg, 767 mmol) and the photocatalyst 2,2‑dimethoxy‑1,2‑diphenylethan‑1‑one (0.40 eq, 19.7 mg, 76.7 mmol) were added to the reaction mixture. Argon was bubbled through the solution for about 20 min. The reaction was conducted under UV light (365 nm) for a duration of 6 hours. The reaction was monitored using TLC. Once the reaction was completed the solvent was removed under reduced pressure. The solid left in the flask was dissolved in water and dialyzed (100‑500 D) against water for 2 days. The water was changed three times a day. The residue was lyophilized. The product (150 mg, 150%) was obtained as a fluffy white solid.

**^1^H NMR** (700 MHz, MeOD, ppm) δ = 8.04 (s, 1H), 4.59 (s, 2H), 3.91 – 3.84 (m, 2H), 3.51 – 3.49 (m, 4H), 2.93 – 2.88 (m, 8H), 2.86 – 2.84 (m, 2H), 2.68 – 2.57 (m, 5H), 2.51 – 2.49 (m, 2H), 2.20 – 2.16 (m, 3H), 2.07 – 2.00 (m, 5H), 1.78 – 1.76 (m, 2H), 1.58 – 1.56 (m, 2H), 1.33 – 1.28 (m, 28H), 0.90 (t, 3H).

**^13^C NMR** (176 MHz, MeOD, ppm) δ = 124.98, 71.56, 70.73, 64.62, 62.53, 51.43, 50.99, 38.19, 33.06, 31.65, 31.07 – 30.15 (m), 29.04, 27.24, 26.17, 25.74, 24.04, 23.72, 19.97, 14.43.

#### pG1-OH

Synthesized according to literature protocol.^[3]^

**^1^H NMR** (500 MHz, MeOD, ppm) δ = 4.26 (p, *J* = 5.7 Hz, 2H), 4.06 – 4.04 (m, 2H), 3.87 – 3.85 (m, 1H), 3.74 – 3.72 (m, 2H), 3.55 – 3.50 (m, 8H), 1.39 (s, 6H), 1.33 (s, 6H).

**^13^C NMR** (151 MHz, MeOD, ppm) δ = 109.2, 74.8, 72.7, 72.6, 72.1, 69.3, 66.3, 48.0, 47.9, 47.7, 47.6, 47.5, 25.8, 24.4.

**HRMS (ESI):** *m*/*z* calcd for C_15_H_28_O_7_Na: 343.1733 [*M*+Na]^+^; found: 343.1787.

#### pG1-N_3_

pG1-OH (1.0 eq, 4.00 g, 12.5 mmol) was dissolved in DCM (50 mL). Under cooling with an ice bath, NEt_3_ (3.5 eq, 6.10 mL, 43.7 mmol) was added slowly. After 1 h of stirring MsCl (1.5 eq, 1.50 mL, 18.8 mmol) was added portion wise. The reaction was carried out overnight at rt. The progress of the reaction was monitored by the thin layer chromatography using hexane/EtOAc. The crude product was extracted with DCM/H_2_O (3 x 100 mL). The combined organic layers were dried over Na_2_SO_4_. The obtained pG1-OMs directly further reacted and dissolved in dry DMF (35 mL). NaN_3_ (3.0 eq, 2.61 g, 39.9 mmol) was added, the reaction mixture was heated up to 80 °C and stirred overnight. The progress of the reaction was monitored by thin layer chromatography using DCM/MeOH. The crude product was extracted with DCM/H_2_O (3 x 100 mL). The combined organic layers were dried over Na_2_SO_4_ and purified with column chromatography using DCM/MeOH as eluent. The product pG1-N_3_ (3.80 g, 85%) was obtained.

**^1^H NMR** (700 MHz, MeOD, ppm) δ = 4.26 – 4.24 (m, 2H), 4.06 – 4.04 (m, 2H), 3.76 – 3.68 (m, 3H), 3.67 – 3.63 (m, 2H), 3.61 – 3.51 (m, 6H), 1.39 (s, 6H), 1.33 (s, 6H).

**^13^C NMR** (176 MHz, MeOD, ppm) δ = 73.8, 73.7, 72.2, 72.1, 72.1, 64.4, 64.3, 62.0, 49.5, 49.3, 49.2, 49.0, 49.0, 48.8, 48.7, 48.5.

**HRMS (ESI):** *m*/*z* calcd for C_15_H_27_N_3_Na O_6_: 343.1798 [*M*+Na]^+^; found: 368.1787.

#### G1-N_3_

pG1-N_3_ (1.0 eq, 3.50 g, 10.1 mmol) was dissolved in MeOH. The acidic resin, Dowex 50WX8 (50w%, 1.75 g) and was added to the reaction mixture. The reaction was carried out overnight at 50 °C and monitored by thin layer chromatography using DCM/MeOH. The resin was removed by filtration, the solvent was removed under reduced pressure, and the product (2.60 g) was obtained with a yield of 94%.

**^1^H NMR** (500 MHz, MeOD, ppm) δ = 3.78 – 3.70 (m, 3H), 3.65 – 3.40 (m, 12H).

**^13^C NMR** (151 MHz, MeOD, ppm) δ = 73.8, 73.7, 72.2, 64.4, 62.0.

**HRMS (ESI):** *m*/*z* calcd for C_9_H_19_N_3_NaO_6_: 288.1172 [*M*+Na]^+^; found: 288.1188.

#### G1-4allyl

NaH (6.0 eq, 1.10 g, 45.2 mmol) was dissolved under dry conditions and ice cooling in DMF (50 mL). G1-N_3_ (1.0 eq, 2.0 g, 7.5 mmol) was added portion wise to the reaction mixture. After 20 min, 3-brompropene (6.0 eq, 5.50 g, 45.2 mmol) was added slowly. The reaction was carried out overnight at 60 °C. The progress of the reaction was monitored by TLC. Once the reaction was completed, the solvent was removed on the rotavapor, and the crude was extracted using DCM/H_2_O. The combined organic layers were dried over Na_2_SO_4_ and purified by column chromatography using hexane/EtOAc. The product (2.80 g) was obtained with a yield of 85%.

**^1^H NMR** (600 MHz, CDCl_3_, ppm) δ = 5.94 – 5.84 (m, 4H), 5.30 – 5.24 (m, 4H), 5.17 – 5.14 (m, 4H), 4.14 – 4.12 (m, 4H), 4.00 – 3.98 (m, 4H), 3.72 – 3.47 (m, 15H).

**^13^C NMR** (151 MHz, CDCl_3_, ppm) δ = 135.2, 134.8, 117.1, 117.0, 116.8, 77.4, 77.2, 77.1, 77.0, 72.5, 71.8, 71.5, 71.4, 71.3, 70.0, 60.7.

**HRMS (ESI):** *m*/*z* calcd for C_21_H_35_N_3_NaO_6_: 448.2424 [*M*+Na]^+^; found: 448.2402.

#### C_12_-4allyl

G1-4allyl (1.0 eq, 1.00 g, 2.82 mmol) and C_12_-alkyne (1.2 eq, 0.63 g, 2.82 mmol) were dissolved in THF (10 mL). CuSO_4_ (0.2 eq, 75.0 mg, 0.47 mmol) and Sodium ascorbate (0.4 eq, 186 mg, 0.94 mmol) were separately dissolved in water (2 mL) and added to the reaction flask. Argon was bubbled through the solution for 15 min. The reaction mixture was carried out at 50 °C overnight. The progress of the reaction was monitored using TLC (cyclohexane/EtOAc). After completion of the reaction, the solvents were removed under reduced pressure, and the residue was extracted using DCM/H_2_O and washed with a saturated solution of EDTA. The combined organic layers were dried over Na_2_SO_4_ and purified by column chromatography using cyclohexane/EtOAc. The product (1.6 g) was obtained in a yield of 93% as a yellowish oil.

**^1^H NMR** (600 MHz, CDCl_3_, ppm) δ = 7.71 (s, 1H), 5.95 – 5.79 (m, 4H), 5.38 – 5.08 (m, 8H), 4.89 – 4.88 (m, 1H), 4.58 (s, 2H), 4.08 – 3.86 (m, 12H), 3.61 – 3.40 (m, 12H), 1.63 – 1.49 (m, 2H), 1.33 – 1.18 (m, 18H), 0.86 (t, *J* = 7.0 Hz, 3H).

**^13^C NMR** (151 MHz, CDCl_3_, ppm) δ = 145.1, 135.1, 134.9, 134.7, 122.8, 117.3, 117.1, 117.1, 72.5, 72.4, 71.7, 71.4, 71.2, 71.0, 70.3, 69.8, 64.4, 60.6, 32.0, 29.8, 29.7, 29.7, 29.6, 29.4, 26.2, 22.8, 14.2.

**HRMS (ESI):** *m*/*z* calcd for C_36_H_63_N_3_NaO_7_: 672.4564 [*M*+Na]^+^; found: 672.4514.

#### C_18_-4allyl

G1-4allyl (1.0 eq, 1.40 g, 3.30 mmol) and C_18_-alkyne (1.2 eq, 1.20 g, 4.00 mmol) were dissolved in THF (10 mL). CuSO_4_ (0.2 eq, 105 mg, 0.7 mmol) and sodium ascorbate (0.4 eq, 261 mg, 1.30 mmol) were separately dissolved in water (3 mL). The mixture was added to the reaction flask. The reaction was carried out at 50 °C overnight and monitored using TLC. After completion of the reaction, the solvents were removed under reduced pressure and the residue was extracted using DCM/H_2_O (3 x 100 mL) and washed with a saturated solution of EDTA. The combined organic layers were dried over Na_2_SO_4_ and purified by column chromatography using cyclohexane/EtOAc. The product (2.20 g) was obtained in a yield of 90% as a yellowish oil.

**^1^H NMR** (400 MHz, CDCl_3_, ppm) δ = δ 7.72 (s, 1H), 5.92 – 5.81 (m, 4H), 5.29 – 5.12 (m, 8H), 4.94 – 4.84 (m, 1H), 4.59 (s, 2H), 4.17 – 3.81 (m, 13H), 3.79 – 3.32 (m, 13H), 1.60 – 1.53 (m, 2H), 1.33 – 1.18 (m, 28H), 0.86 (t, *J* = 6.8 Hz, 3H).

**^13^C NMR** (101 MHz, CDCl_3_, ppm) δ = 145.0, 135.0, 134.7, 122.8, 117.2, 117.1, 77.5, 77.2, 76.9, 76.8, 72.5, 71.7, 71.4, 71.1, 70.3, 69.8, 64.4, 60.6, 32.0, 29.8, 29.7, 29.5, 26.3, 22.8, 14.3.

**MS (ESI)** m/z = 756.5469; [M+Na]^+^ (calcd. For: C_42_H_75_N_3_NaO_7_: 756.5503).

#### C_12_-4S

C_12_-4allyl (1.0 eq, 50.0 mg, 0.08 mmol) was dissolved in MeOH (20 mL). Sodium 3-mercaptopropyl sulfonate (8.0 eq, 120 mg, 0.62 mmol) and DMPA (0.4 eq, 7.90 mg, 0.04 mmol) were added to the reaction mixture. The reaction was carried out under UV-light (365 nm) for 3 h. The progress of the reaction was monitored by TLC. After completion of the reaction, the solvent was removed, and the crude was dissolved in water and dialyzed (100-500 D) against water for 2 days (changing the water thrice a day). The residue was lyophilized, and the product (95 mg) was obtained in a yield of 91% as a fluffy white solid.

**^1^H NMR** (700 MHz, MeOD, ppm) δ = 8.08 (s, 1H), 4.59 (s, 2H), 4.00 – 3.92 (m, 3H), 3.66 – 3.38 (m, 15H), 3.00 – 2.91 (m, 14H), 2.85 (t, *J* = 7.2 Hz, 3H) 2.66 – 2.64 (m, 8H),2.60 – 2.58 (m, 4 H) 2.26 – 2.17 (m, 3H), 2.11 – 1.95 (m, 11H), 1.82 – 1.78 (m, 4H), 1.60 – 1.58 (m, 2H), 1.36 – 1.23 (m, 18H), 0.90 (m, 3H).

**^13^C NMR** (176 MHz, MeOD, ppm) δ = 144.6, 125.1, 79.1, 72.2, 71.7, 71.4, 70.9, 69.7, 64.7, 62.5, 38.2, 33.0, 31.7, 31.0, 30.7, 30.6, 30.4, 29.1, 27.2, 26.2, 25.7, 24.0, 23.7, 19.9, 14.4.

**HRMS (ESI):** *m*/*z* calcd for C_48_H_91_N_3_Na_3_O_19_S_8_: 1338.3706 [*M*]^–^; found: 1338.5309.

#### C_18_-4S

C_18_-dTG-allyl (1.0 eq, 500 mg, 0.7 mmol) was dissolved in MeOH (20 mL). sodium 3-mercaptopropyl sulfonate (6.0 eq, 794 mg, 4.1 mmol) and DMPA (0.4 eq, 70 mg, 0.3 mmol) were added to the reaction mixture. The reaction was carried out under UV-light (365 nm) for 6 h. The progress of the reaction was monitored by TLC. After completion of the reaction, the solvent was removed, and the crude was dissolved in water and dialyzed (100-500 D) against water for 2 days (changing the water thrice a day). The residue was lyophilized, and the product (790 mg) was obtained in a yield of 91% as a fluffy white solid.

**^1^H NMR** (700 MHz, MeOD, ppm) δ = 8.08 (s, 1H), 4.60 (s, 2H), 4.03 – 3.86 (m, 4H), 3.69 – 3.36 (m, 20H), 2.99 – 2.97 (m, 1H), 2.96 – 2.88 (m, 8H), 2.66 – 2.58 (m, 14H), 2.30 – 2.02 (m, 8H), 1.85 – 1.75 (m, 8H), 1.62 – 1.58 (m, 2H), 1.35 – 1.25 (m, 32H), 0.90 (t, *J*=7.0 Hz, 3H).

**^13^C NMR** (176 MHz, MeOD, ppm) δ = 145.8, 125.0, 79.1, 79.1, 72.3, 72.2, 71.7, 71.5, 71.4, 70.9, 69.7, 64.7, 62.6, 51.5, 33.1, 31.7, 31.7, 31.1, 30.8, 30.6, 30.5, 29.2, 29.2, 27.3, 26.2, 23.7, 14.4.

**HRMS (ESI):** *m*/*z* calcd for C_54_H_103_N_3_Na_3_O_19_S_8_: 1422.4645 [*M*]^–^; found: 1422.4670.

#### lTG-allyl

NaH (8.0 eq, 0.22 g, 9.05 mmol) was dissolved under dry conditions and ice cooling in dry DMF (20 mL). lTG-N_3_, synthesized following the literature^[4]^, (1.0 eq, 0.30 g, 1.13 mmol) dissolved in dry DMF (2mL) was slowly added to the reaction mixture. After 20 min of stirring, 3-Brompropene (8.0 eq, 1.09 g, 9.05 mmol) was added dropwise. The reaction was carried out overnight at 60 °C. The progress of the reaction was monitored by TLC (20% EtOAc in cyclohexane). After completion of the reaction, the solvent was removed on the rotavapor, and the crude was extracted using DCM/H_2_O. The combined organic layers were dried over Na_2_SO_4_ and purified by column chromatography using cyclohexane/EtOAc. The product was obtained with a yield of 87%.

**^1^H NMR** (500 MHz, CDCl_3_, ppm) δ = 5.96 – 5.85 (m, 4H), 5.28 – 5.15 (m, 8H), 4.16 – 4.08 (m, 6H), 4.03 – 3.99 (m, 2H), 3.69 – 3.63 (m, 3H), 3.59 – 3.47 (m, 10H), 3.39 – 3.31 (m, 2H).

**^13^C NMR** (151 MHz, CDCl_3_, ppm) δ 135.3, 134.9, 117.1, 77.4, 77.2, 77.0, 72.5, 71.7, 71.5, 54.7.

**HRMS (ESI):** *m*/*z* calcd for C_21_H_35_N_3_NaO_23_: 448.2526 [*M*+Na]^+^; found: 448.2402.

#### C_18_-lTG-4allyl

lTG-N_3_-allyl (1.0 eq, 100 mg, 235 µmol) and C_18_-alkyne (1.2 eq, 87.0 mg, 282 µmol) were dissolved in DMF (10 mL) stirred well. Afterwards Cu(I)Ac (0.4 eq, 10.1 mg, 94.0 µmol) was added to the reaction flask. Argon was bubbled for 20 min through the solution. The reaction was stirred at 50 °C overnight. The reaction was monitored using TLC (40% EtOAc in cyclohexane). After completion of the reaction, the solvents were removed under reduced pressure and the residue was extracted using DCM/H_2_O and washed with a saturated solution of EDTA. The combined organic layers were dried over Na_2_SO_4_ and purified by column chromatography using cyclohexane/EtOAc. The product was obtained in a yield of 40% as a yellowish oil.

**^1^H NMR** (500 MHz, CDCl_3_, ppm) δ 7.65 (s, 1H), 5.90 – 5.84 (m, 3H), 5.77 – 5.69 (m, 1H), 5.38 – 5.17 (m, 4H), 5.22 – 5.07 (m, 4H), 4.61 (s, 2H), 4.59 – 4.57 (m, 1H), 4.41 – 4.35 (m, 1H), 4.18 – 4.09 (m, 4H), 4.09 – 3.95 (m, 3H), 3.93 – 3.82 (m, 2H), 3.73 – 3.64 (m, 2H), 3.68 – 3.34 (m, 12H), 1.61 – 1.54 (m, 2H), 1.33 – 1.20 (m, 30H), 0.87 (t, *J* = 6.9 Hz, 3H).

**HRMS (ESI):** *m*/*z* calcd C_42_H_75_N_3_NaO_7_: 756.5503 [*M*+Na]^+^; found: 756.5469.

#### C_18_-lTG-4S

C_18_-lTG-4allyl (1.0 eq, 27.0 mg, 0.04 mmol) was dissolved in MeOH (20 mL). sodium 3-mercaptopropyl sulfonate (8.0 eq, 57.2 mg, 0.29 mmol) and DMPA (0.4 eq, 3.80 mg, 0.02 mmol) were added to the reaction mixture. The reaction was carried out under UV-light (365 nm) for 3 h. The progress of the reaction was monitored by TLC. After completion of the reaction, the solvent was removed, and the crude was dissolved in water and dialyzed (100-500 D) against water for 2 days (changing the water thrice a day). The residue was lyophilized, and the product (50.0 mg) was obtained in a yield of 91% as a fluffy white solid.

**^1^H NMR** (700 MHz, MeOD, ppm) δ 4.74 – 4.52 (m, 4H), 4.23 – 3.86 (m, 4H), 3.77 – 3.43 (m, 15H), 2.96 – 2.87 (m, 14H), 2.67 – 2.61 (m, 12H), 2.21 – 2.16 (m, 2H), 2.13 – 1.95 (m, 14H), 1.84 (s, 2H), 1.76 – 1.69 (m, 1H), 1.59 (s, 2H), 1.25 – 1.18 (s, 30H), 0.90 (t, *J* = 7.0 Hz, 3H).

**^13^C NMR** (176 MHz, MeOD, ppm) δ 141.5, 123.43, 72.2, 69.7, 38.2, 33.1, 31.7, 30.8, 30.6, 29.2, 27.2, 26.2, 25.7, 24.0, 23.7, 14.4.

**HRMS (ESI):** *m*/*z* calcd for C_54_H_103_N_3_Na_3_O_19_S_8_: 1422.4645 [*M*]^–^; found: 1422.4702.

#### **pG2-OH**

Synthesized according to literature protocol.^[3]^

**^1^H NMR** (600 MHz, MeOD, ppm) δ 4.29 – 4.21 (m, 4H), 4.06 – 4.04 (m, 4H), 3.84 – 3.79 (m, 1H), 3.75 – 3.71 (m, 4H), 3.70 – 3.64 (m, 4H), 3.63 – 3.49 (m, 18H), 1.39 (s, 12H), 1.33 (s, 12H) ppm.

**^13^C NMR** (151 MHz, MeOD, ppm) δ 110.4, 79.7, 76.0, 73.3, 72.7, 72.3, 72.3, 72.2, 67.46, 27.0, 25.5.

#### pG2-N_3_

pG2-OH (1.0 eq, 4.00 g, 5.74 mmol) was dissolved in DCM (200 mL). Under cooling with an ice bath, NEt_3_ (3.5 eq, 2.03 g, 20.1 mmol) was added slowly. After 30 min of stirring MsCl (1.5 eq, 0.99 g, 8.61 mmol) was added portion wise. The reaction was stirred overnight at rt. The progress of the reaction was monitored by the thin layer chromatography using cyclohexane/EtOAc. The crude product was extracted with DCM/H_2_O. The combined organic layers were dried over Na_2_SO_4_. The crude product was dissolved in DMF (50 mL). NaN_3_ (3.5 eq, 1.25 g, 20.1 mmol) was added, the reaction mixture was heated up to 80 °C and stirred overnight. The progress of the reaction was monitored by thin layer chromatography using DCM/MeOH. The crude product was extracted with DCM/H_2_O. The combined organic layers were dried with Na_2_SO_4_ and purified with column chromatography using DCM/MeOH. The product pG2-N_3_ (3.30 g, 80%) was obtained.

**^1^H NMR** (500 MHz, MeOD, ppm) δ 4.30 – 4.21 (m, 4H), 4.15 – 4.04 (m, 5H), 3.80 – 3.50 (m, 26H), 1.39 (s, 12H), 1.33 (s, 12H).

**^13^C NMR** (176 MHz, MeOD, ppm) δ 110.6, 80.0, 76.1, 73.4, 72.5, 72.5, 72.4, 71.2, 67.5, 62.5, 27.1, 25.7.

**HRMS (ESI):** *m*/*z* calcd C_33_H_59_N_3_NaO_14_: 744.3997 [*M+Na*]^+^; found: 744.3910.

#### G2-allyl

NaH (12 eq, 0.52 g, 21.4 mmol) was dissolved under dry conditions and ice cooling in DMF (50 mL). G2-N_3_ (1.0 eq, 1.00 g, 1.78 mmol) was added portion wise to the reaction mixture. After 20 min, 3-brompropene (12 eq, 2.59 g, 21.4 mmol) was added slowly. The reaction was carried out overnight at 60 °C. The progress of the reaction was monitored by TLC. Once the reaction was completed, the reaction was quenched with *iso*-propanol The solvent was removed on the rotavapor, and the crude was extracted using DCM/H_2_O. The combined organic layers were dried over Na_2_SO_4_ and purified by column chromatography using cyclohexane/EtOAc. The product (1.32 g) was obtained with a yield of 84%.

**^1^H NMR** (500 MHz, CDCl_3_, ppm) δ 5.96 – 5.85 (m, 8H), 5.32 – 5.24 (m, 8H), 5.18 – 5.14 (m, 8H), 4.21 – 4.07 (m, 8H), 4.01 – 3.99 (m, 8H), 3.80 – 3.44 (m, 35H).

**^13^C NMR** (101 MHz, CDCl_3_, ppm) δ 135.0, 134.6, 116.7, 72.2, 71.5, 71.2, 69.9.

**HRMS (ESI):** *m*/*z* calcd C_45_H_75_N_3_NaO_14_: 904.5249 [*M+*Na]^+^; found: 904.5232.

#### C18-8allyl

G2-8allyl (1.0 eq, 1.00 g, 1.13 mmol) and C_18_-alkyne (1.2 eq, 0.42 g, 1.36 mmol) were dissolved in THF (100 mL). CuSO_4_ (0.2 eq, 0.04 g, 0.02 mmol) and sodium ascorbate (0.4 eq, 0.09 mg, 0.05 mmol) were separately dissolved in water (1 mL). The mixture was added to the reaction flask. The reaction was carried out at 50 °C overnight and monitored using TLC. After completion of the reaction, the solvents were removed under reduced pressure and the residue was extracted using DCM/H_2_O (3 x 100 mL) and washed with a saturated solution of EDTA. The combined organic layers were dried over Na_2_SO_4_ and purified by column chromatography using cyclohexane/EtOAc. The product (0.85 g) was obtained in a yield of 75% as a yellowish oil.

**^1^H NMR** (400 MHz, CDCl_3_, ppm) δ 7.94 – 7.82 (m, 1H), 5.74 – 5.65 (m, 8H), 5.09 – 5.03 (m, 8H), 4.96 – 4.87 (m, 8H), 4.36 (s, 2H), 4.00 – 3.66 (m, 24H), 3.45 – 3.25 (m, 31H), 1.42 – 1.36 (m, 2H), 1.14 – 1.04 (m, 28H), 0.69 (t, *J* = 6.7 Hz, 3H).

**^13^C NMR** (101 MHz, CDCl_3_, ppm) δ 136.59, 136.14, 117.18, 117.05, 73.30, 72.52, 72.23, 71.10, 49.65, 49.44, 49.23, 49.01, 48.80, 48.59, 48.37, 30.81.

**HRMS (ESI):** *m*/*z* calcd C_66_H_115_N_3_NaO_15_: 1112.8226 [*M+*Na]^+^; found: 1112.8176.

#### C18-8S

C_18_-8allyl (1.0 eq, 90.0 mg, 0.08 mmol) was dissolved in MeOH (20 mL). sodium 3-mercaptopropyl sulfonate (12 eq, 180 mg, 0.91 mmol) and DMPA (0.4 eq, 7.90 mg, 0.03 mmol) were added to the reaction mixture. The reaction was carried out under UV-light (365 nm) for 3 h. The progress of the reaction was monitored by TLC. After completion of the reaction, the solvent was removed, and the crude was dissolved in water and dialyzed (100-500 D) against water for 2 days (changing the water thrice a day). The residue was lyophilized, and the product (170 mg) was obtained in a yield of 82% as a fluffy white solid.

**^1^H NMR** (500 MHz, MeOD, ppm) δ 8.14 (s, 1H), 4.12 – 4.08 (m, 3H), 3.68 – 3.51 (m, 42H), 3.03 – 2.82 (m, 35H), 2.66 – 2.61 (m, 25H), 2.18 – 2.15 (m, 6H), 2.06 – 2.03 (m, 16H), 1.84 – 1.83 (m, 10H), 1.59 (t, *J* = 7.2 Hz, 2H), 1.31 – 1.28 (s, 28H), 0.89 (t, *J* = 6.8 Hz, 3H).

**HRMS (ESI):** *m*/*z* calcd C_90_H_171_N_3_Na_7_O_39_S_16_: 2590.6349 [*M-*Na]^-^; found: 2590.6402.

#### C18-pG1

pG1-N3 (1.0 eq, 1.00 g, 2.90 mol) and C_18_-alkyne (1.2 eq, 0.82 g, 3.20 mmol) were dissolved in DMF (100 mL) stirred well. Afterwards CuSO_4_ (0.2 eq, 90.0 mg, 0.58 mmol) and sodium ascorbate (0.4 eq, 228 mg, 1.16 mmol) were premixed in water (0.5 mL) and added to the reaction flask. Argon was bubbled for 20 min through the solution. The reaction was stirred at 50 °C overnight. The reaction was monitored using TLC (40% EtOAc in cyclohexane). After completion of the reaction, the solvents were removed under reduced pressure, and the residue was extracted using DCM/H_2_O and washed with a saturated solution of EDTA. The combined organic layers were dried over Na_2_SO_4_ and purified by column chromatography using cyclohexane/EtOAc. The product was obtained in a yield of 85% as a yellowish oil.

**^1^H NMR** (500 MHz, MeOD, ppm) δ 8.06 (s, 1H), 5.05 – 4.92 (m, 1H), 4.57 (s, 2H), 4.21 – 4.16 (m, 2H), 3.99 – 3.96 (m, 6H), 3.66 – 3.58 (m, 2H), 3.57 – 3.41 (m, 6H), 1.72 – 1.50 (m, 2H), 1.43 – 1.18 (m, 42H), 0.96 – 0.86 (m, 3H).

#### C18-4OH

C18-pG1 (1.5 g) was dissolved in MeOH (50 mL), Dowex 50wx8 (0.75 g, 50w%) was added. The reaction was stirred at 50 °C overnight. The reaction was monitored using TLC (40% EtOAc in cyclohexane). After completion of the reaction the resin was removed by filtration, and the product was concentrated using the rotavapor. The product was obtained with 95% as a colourless oil and directly used for further reaction.

**^1^H NMR** (700 MHz, MeOD, ppm) δ 8.09 (s, 1H), 5.03 – 4.99 (m, 1H), 4.57 (s, 2H), 3.99 – 3.90 (m, 4H), 3.75 – 3.67 (m, 2H), 3.57 – 3.43 (m, 10H), 1.63 – 1.55 (m, 2H), 1.29 (s, 30H), 0.90 (t, *J* = 7.1 Hz, 3H).

**^13^C NMR** (176 MHz, MeOD, ppm) δ 145.74, 125.05, 73.80, 73.71, 72.12, 72.06, 71.72, 71.30, 71.28, 64.64, 64.37, 64.24, 64.22, 62.39, 33.07, 30.78, 30.74, 30.68, 30.60, 30.47, 27.21, 23.73, 14.44.

#### C_18_-3OH-N_3_

C_18_-4OH (1.0 eq, 1.00 g, 1.74 mmol) was dissolved in DCM (100 mL). Under cooling with an ice bath, NEt_3_ (2.0 eq, 0.48 mL, 3.49 mmol) was added slowly. After 1 h of stirring MsCl (1.5 eq, 122 µL, 1.57 mmol) was added portion wise. The reaction was carried out overnight at rt. The progress of the reaction was monitored by the thin layer chromatography using DCM/MeOH. The obtained C_18_-3OH-OMs directly further reacted and dissolved in dry DMF (100 mL). NaN_3_ (3.0 eq, 200 mg, 3.07 mmol) was added, the reaction mixture was heated up to 80 °C and stirred overnight. The progress of the reaction was monitored by thin layer chromatography using DCM/MeOH. After the removal of the solvent, the crude product was purified with column chromatography using DCM/MeOH as eluent. The product C_18_-3OH-N_3_ (0.9 g, 80%) was obtained.

**^1^H NMR** (400 MHz, CDCl_3_, ppm) δ 7.79 (s, 1H), 4.92 (p, *J* = 5.6 Hz, 1H), 4.54 (s, 2H), 3.90 (q, *J* = 5.0 Hz, 4H), 3.79 (q, *J* = 5.1 Hz, 2H), 3.58 – 3.43 (m, 10H), 1.57 (q, *J* = 7.0 Hz, 2H), 1.22 (d, *J* = 1.8 Hz, 30H), 0.88 – 0.82 (m, 3H).

#### C_18_-3allyl-N_3_

NaH (8.0 eq, 0.45 g, 10.7 mmol) was dissolved under dry conditions and ice cooling in DMF (100 mL). C_18_-3OH-N_3_ (1.0 eq, 0.80 g, 1.34 mmol) was added to the reaction mixture. After 20 min, 3-brompropene (8.0 eq, 0.95 mL, 10.7 mmol) was added slowly. The reaction was carried out overnight at 50 °C. The progress of the reaction was monitored by TLC. Once the reaction was completed, the reaction was quenched using isopropanol. The solvent was removed on the rotavapor, and the crude was extracted using DCM/H_2_O. The combined organic layers were dried over Na_2_SO_4_ and purified by column chromatography using Hexane/EtOAc. The product (0.75 g) was obtained with a yield of 78%.

**^1^H NMR** (400 MHz, CDCl_3_, ppm) δ 7.69 (s, 1H), 5.92 – 5.81 (m, 3H), 5.40 – 5.05 (m, 6H), 4.91 – 4.87 (m, 1H), 4.58 (s, 2H), 4.18 – 3.78 (m, 10H), 3.66 – 3.34 (m, 10H), 3.33 – 3.15 (m, 2H), 1.61 – 1.58 (m, 2H), 1.23 (s, 30H), 0.86 (t, *J* = 6.8 Hz, 3H).

#### C_18_-3S-NH_2_

C_18_-3allyl-N_3_ (1.0 eq, 0.50 g, 0.70 mmol) was dissolved in THF/water (1:1, 50 mL). TCEP (2.0 eq, 0.04 g, 0.14 mmol) was added to the reaction mixture. The progress of the reaction was monitored by thin layer chromatography using cyclohexane/EtOAc (3% NEt_3_). The reaction mixture was neutralized using a saturated NaHCO_3_ solution and extracted using water/DCM (3 x 100 mL). The crude product was directly used for further reactions. C_18_-3allyl-NH_2_ (1.0 eq, 50.0 mg, 0.07 mmol) was dissolved in MeOH (20 mL). Sodium 3-mercaptopropyl sulfonate (6.0 eq, 77.0 mg, 0.43 mmol) and DMPA (0.4 eq, 7.40 mg, 0.03 mmol) were added to the reaction mixture. The reaction was carried out under UV-light (365 nm) for 3 h. The progress of the reaction was monitored by TLC. After completion of the reaction, the solvent was removed, and the crude was dissolved in water and dialyzed (100-500 D) against water for 2 days (changing the water thrice a day). The residue was lyophilized, and the product (100 mg) was obtained in a yield of 79% as a fluffy white solid.

**^1^H NMR** (500 MHz, MeOD, ppm) δ 8.09 (s, 1H), 5.32 – 4.97 (m, 2H), 4.65 – 4.52 (m, 2H), 3.96 (s, 5H), 3.85 – 3.24 (m, 15H), 2.99 – 2.89 (m, 9H), 2.85 (t, *J* = 7.1 Hz, 2H), 2.74 – 2.51 (m, 8H), 2.40 – 1.91 (m, 10H), 1.80 (q, *J* = 6.3 Hz, 2H), 1.55 – 1.53 (m, 2H), 1.29 (s, 30H), 0.90 (t, *J* = 6.8 Hz, 3H).

#### C_18_-3S-Cy3

C_18_-3S-NH_2_ (1.50 eq, 12.3 mg, 7.01 µmol) was dissolved in dry DMF (10 mL). DIPEA (3.0 eq, 2.44 µL, 14.0 µmol) was added to the reaction mixture. Lastly, Cy3-NHS (provided from Kai Licha) (1.0 eq, 3.57 mg, 4.67 µmol) was added to the reaction. The reaction was carried out overnight at 40 °C. The progress of the reaction was monitored by reverse phase thin layer chromatography using ACN/H_2_O. After completion of the reaction, the solvent was removed the crude was dissolved in water and dialyzed (100-500 D) against water for 2 days (changing the water thrice a day). The residue was lyophilized, and the product (6.0 mg) was obtained in a yield of 70% as a fluffy red solid. From the 1H-NMR we can estimate the degree of functionalization to 50%.

**^1^H NMR** (600 MHz, MeOD, ppm) δ 8.59 – 8.55 (m, 0.5H), 8.13 – 8.06 (m, 1H), 7.60 – 7.33 (m, 3H), 6.81 – 6.46 (m, 0.5H), 4.59 (s, 1H), 4.37 – 4.15 (m, 2H), 3.96 (s, 3H), 3.56 - 3.50 (m, 10H), 3.04 – 2.82 (m, 16H), 2.74 – 2.53 (m, 4H), 2.32 – 2.12 (m, 5H), 2.12 – 1.90 (m, 9H), 1.84 – 1.71 (m, 8H), 1.58 (s, 2H), 1.43 – 1.13 (m, 30H), 0.90 (t, *J* = 6.9 Hz, 3H).

## 1.2 Physicochemical Properties of Surfactants

Table S2: Overview of physicochemical properties of sulfonated surfactants. The diameter of the self-assembled aggregates (in nanometer) derived by dynamic light scattering (Figure S1). Zetapotential in mV and critical micelle concentration derived by DLS measurements in µM or mg/mL (Figure S2). All samples have been measured in PBS (10 mM).

| **Surfactant** | **DLS [d. nm]** | **Zeta [mV]** | **CMC [µM]** | **CMC [mg/mL]** |
| --- | --- | --- | --- | --- |
| C_12_-2S | 170 | -47.96 ± 6.54 | 100 | 0.13 |
| C_18_-2S | 6 | -42.45 ± 2.68 | 6 | 0.007 |
| C_12_-4S | 110 | -43.72 ± 0.35 | 130 | 0.09 |
| C_18_-4S | 5 | -44.23 ± 0.99 | 40 | 0.03 |
| C_18_-lTG-4S | 5 | -43.36 ± 1.98 | 35 | 0.03 |
| C18-8S | 18 | -39.17 ± 1.22 | 80 | 0.03 |
| SDS | 370 | -20.42 ± 0.99 | 1350 | 4.69 |

## 1.3 Dynamic Light Scattering (DLS)

Figure S1: Dynamic light scattering (DLS) of sulfonated surfactants. Number based population plotted over size of particles in d. nm. Populations of nanometer sized micelles (6-370 nm) can be observed. All samples have been measured in PBS (10 mM) at a fixed surfactant concentration of 1 mM.

## 1.4 Critical micelle concentration

Figure S2: Critical micelle concentration (CMC), derived by DLS measurements. Two linear fits were applied to the plots of the derived count rate and the surfactant concentration. All samples have been measured in PBS (10 mM).

## 1.5 Co-assembly Investigations

Figure S3: UV-Vis spectra of BTA co-assemblies. Morphological change over the addition of the different surfactant ratios. All samples have been measured in PBS at a constant BTA concentration of 250 µM and variable molar equivalents of surfactant.

Figure S4: UV-Vis of BTA co-assemblies with different surfactants, showing the maximum absorbance for the different molar equivalents of the different surfactants form the spectra of Figure S3. Morphological transitions can be correlated to the change in absorbance from a peak maximum at 212 nm to lower wavelengths. Not in all cases is clear jump visible like in case of SDS (e), suggesting smooth transitions between the different self-assembled aggregates.

## 1.6 Cryo-TEM

Figure S5: Cryo-TEM micrographs of BTA and BTA co-assemblies with C18-4S. A constant BTA concentration of 0.5 mM and different molar equivalents of C18-4S was used. All samples were prepared in PBS. In case of pure BTA, classical double helical fibers can be observed, as reported in previous investigations.^[5]^ In presence of 0.2 eq of C18-4S a dense network of fibers is visible, indicating that the surfactant does not disrupt the BTA fiber at this ratio. At 1.0 eq only short fibers and micelles probably containing both, BTA and surfactant are present. This ratio of surfactant seems to almost fully solubilize the BTA monomers in small co-micelles, which are hardly visible. Scale bar in the overview micrographs corresponds to 200 nm and the magnifications to 50 nm.

## 1.7 TIRF microscopy

Figure S6: Total internal reflection fluorescence (TIRF) microscopy of BTA co-assembly with 0.2 equivalents of C_18_-4S. (A) Cy3 channel, (B) Cy5 channel and (C) merged channel. Sample of 5 µM BTA (5 mol% BTA-Cy5^[6]^) and 0.2 molar equivalents of C_18_-4S (5 mol% C_18_-3S-Cy3). Scale bar = 10 µm and 2 µm. Colocalization of dye labeled BTA and surfactant in fibrous assemblies.

Figure S7: Total internal reflection fluorescence (TIRF) microscopy of BTA co-assembly with 1.0 equivalents of C_18_-4S. (A) Cy3 channel, (B) Cy5 channel and (C) merged channel. Sample of 5 µM BTA (5 mol% BTA-Cy5) and 0.2 molar equivalents of C_18_-4S (5 mol% C_18_-3S-Cy3). Scale bar = 10 µm. No observation of any larger assemblies containing dye labeled BTA and surfactant.

## 1.8 Cell viability

Figure S8: Cell viability assessment using CCK-8 on Vero E6 cells incubated for 48 h with BTA. No decrease in cell viability up to 2 mM, therefore no CC‑50 value (cytotoxic concentration 50%) can be estimated.

Figure S9: Cell viability assessment using CCK-8 on Vero E6 cells incubated for 48 h with the surfactants. (A-C) Sigmoidal fitted inhibition curves of the different surfactants using dose-response inhibition fit curve. (D) CC‑50 values (cytotoxic concentration 50%) of the different surfactants.

Figure S10: Cell viability assessment of methylcellulose (MC, 2 w%) using CCK-8 on Vero E6 cells incubated for 48 h with and without the surfactants. No toxicity for pure methylcellulose. Same CC-50 values for mixtures with C_12_-4S (>2 mM) and C_18_-4S (1.8 ± 0.1 mM) as for the pure surfactants were obtained. No indication that viscosity impacts on cell viability. Only intercalation into the hydrophobic pocket of the BTA reduces cell toxicity.

## 1.9 Pre-Infection Inhibition of HSV-1

Figure S11: Pre-Infection Inhibition assay of HSV-1 on Vero-E6 cells incubated for 48 h. Two different experimental setups. (Triangle) BTA applied to cells and preincubated for 45 min before the virus is added, creating a gel barrier and avoiding infection up to half maximal inhibitory concentration (IC-50) of 0.42 ± 0.02 mM. (Circle) BTA and virus are preincubated for 45 min leading to full infection at all concentrations, indicating that there is no inhibitory effect of BTA.

Figure S12: Pre-Infection Inhibition assay of HSV-1 on Vero-E6 cells incubated for 48 h. Surfactants solution and virus were preincubated for 45 min before addition to confluent layer of Vero E6 cells. (A-C) Sigmoidal fitted inhibition curves of the different surfactants using dose-response inhibition fit curve. (D) IC-50 values of the different surfactants in micromolar concentration.

Figure S13: Pre-Infection Inhibition assay of HSV-1 on Vero-E6 cells incubated for 48 h. BTA coassemblies with 0.2 eq of surfactant and virus were preincubated for 45 min before addition to confluent layer of Vero E6 cells. (A-C) Sigmoidal fitted inhibition curves of the different co-assemblies using dose-response inhibition fit curves. (D) IC-50 values of the different co-assemblies in micromolar concentration.

Table S3: Cell viability (CC-50) and HSV-1 inhibition (IC-50) of sulfonated *SupraVir* surfactants. Comparison between surfactant itself and BTA – surfactant co-assemblies with 0.2 equivalents. *Inhibition per charge is calculated by the IC-50/#sulfonate per surfactant. **Factor of activity is calculated by the IC-50 (surfactant)/ IC-50 (Co-assembly).

| **Surfactant** | **CC-50**  **Cells**  **[mM]** | **IC-50**  **HSV-1**  **[µM]** | **Inhibition per charge [µM]** | **IC-50 HSV-1**  **Co-assembly [µM]** | **Inhibition per charge***  **[µM]** | **Factor of Activity****  **[-]** |
| --- | --- | --- | --- | --- | --- | --- |
| **C12-2S** | 1.90 ± 0.13 | 540 ± 29 | 270 | 200 ± 25 | 100 | 2.7 |
| **C18-2S** | 0.77 ± 0.12 | 180 ± 11 | 90 | 150 ± 8 | 75 | 1.2 |
| **C12-4S** | >2.00 | 480 ± 70 | 120 | 140 ± 9 | 35 | 3.4 |
| **C18-4S** | 1.80 ± 0.12 | 120 ± 27 | 30 | 50 ± 2 | 12.5 | 2.4 |
| **C18-lTG-4S** | 0.80 ± 0.06 | 140 ± 10 | 35 | 90 ± 5 | 22.5 | 1.6 |
| **C18-8S** | 1.90 ± 0.11 | 260 ± 60 | 32.5 | 130 ± 8 | 16.25 | 2.0 |
| **SDS** | 0.30 ± 0.01 | 340 ± 20 | 340 | 170 ± 19 | 170 | 2.0 |

# 2. Literature

[1]	Y. Kerkhoff, S. Wedepohl, C. Nie, V. Ahmadi, R. Haag, S. Block, *MethodsX* **2022**, *9*, 101834.

[2]	N. Hanheiser, Y. Jiang, C. Zoister, M. Dimde, K. Achazi, C. Nie, Y. Li, R. Haag, A. K. Singh, *Angewandte Chemie International Edition* **2025**, *64*, e202425069.

[3]	M. Wyszogrodzka, R. Haag, *Chem* **2008**, *14*, 9202–14.

[4]	A. K. Singh, M. Seewald, B. Schade, C. Zoister, R. Haag, L. H. Urner, *Commun Chem* **2025**, *8*, 1–11.

[5]	R. P. M. Lafleur, S. Herziger, S. M. C. Schoenmakers, A. D. A. Keizer, J. Jahzerah, B. N. S. Thota, L. Su, P. H. H. Bomans, N. A. J. M. Sommerdijk, A. R. A. Palmans, R. Haag, H. Friedrich, C. Böttcher, E. W. Meijer, *J. Am. Chem. Soc.* **2020**, *142*, 17644–17652.

[6]	L. Albertazzi, D. van der Zwaag, C. M. A. Leenders, R. Fitzner, R. W. van der Hofstad, E. W. Meijer, *Science* **2014**, *344*, 491–495.

# 3. Appendix

## 3.1 NMR

#### Figure S14: ^1^H NMR of C12-alkyne.

#### Figure S15: ^13^C NMR of C12-alkyne.

#### Figure S16: ^1^H NMR of C18-alkyne.

#### Figure S17: ^13^C NMR of C18-alkyne.

#### Figure S18: ^1^H NMR of G0-OH.

#### Figure S19: ^13^C NMR of G0-OH.

#### Figure S20: ^1^H NMR of G0-N3-allyl.

#### Figure S21: ^13^C NMR of G0-N3-allyl.

#### Figure S22: ^1^H NMR of C12-2allyl

#### Figure S23: ^13^C NMR of C12-2allyl.

#### Figure S24: ^1^H NMR of C18-2allyl.

#### Figure S25: ^13^C NMR of C18-2allyl.

#### Figure S26: ^1^H NMR of C12-2S.

#### Figure S27: ^13^C NMR of C12-2S.

#### Figure S28: ^1^H NMR of C18-2S.

#### Figure S29: ^13^C NMR of C18-2S.

#### Figure S30: ^1^H NMR of pG1-OH.

#### Figure S31: ^13^C NMR of pG1-OH.

#### Figure S32: ^1^H NMR of pG1-N_3_.

#### Figure S33: ^13^C NMR of pG1-N_3_.

#### Figure S34: ^1^H NMR of G1-N_3_.

#### Figure S35: ^13^C NMR of G1-N_3_.

#### Figure S36: ^1^H NMR of G1-N_3_-allyl.

#### Figure S37: ^13^C NMR of G1-N_3_-allyl.

 Figure S38: ^1^H NMR of C12-4allyl.

 Figure S39: ^13^C NMR of C12-4allyl

 Figure S40: ^1^H NMR of C12-4S.

 Figure S41: ^13^C NMR of C12-4S.

#### Figure S42: ^1^H NMR of C_18_-4allyl.

#### Figure S43: ^13^C NMR of C_18_-4allyl.

#### Figure S44: ^1^H NMR of C_18_‑4S.

#### Figure S45: ^13^C NMR of C18‑4S.

#### Figure S46: ^1^H NMR of lTG-4allyl.

#### Figure S47: ^13^C NMR of lTG-4allyl.

#### Figure S48: ^1^H NMR of C18-lTG-4allyl.

#### Figure S49: ^1^H NMR of lTG-4S.

#### Figure S50: ^13^C NMR of lTG-4S.

Figure S51: 1H NMR of pG2-OH.

#### Figure S52: ^13^C NMR of pG2-OH.

#### Figure S53: ^1^H NMR of pG2-N3.

#### Figure S54: ^13^C NMR of pG2-N3.

#### Figure S55: ^1^H NMR of G2-N3-8allyl.

#### Figure S56: ^13^C NMR of G2-N3-8allyl.

#### Figure S57: ^1^H NMR of C18-8allyl.

#### Figure S58: ^13^C NMR of C18-8allyl.

#### Figure S59: ^1^H NMR of C18-8S.

#### Figure S60: ^1^H NMR of C18-pG1.

#### Figure S61: ^1^H NMR of C18-4OH.

#### Figure S62: ^13^C NMR of C18-4OH.

#### Figure S63: ^1^H NMR of C18-3OH-N3.

#### Figure S64: ^1^H NMR of C18-3allyl-N3.

#### Figure S65: ^1^H NMR of C18-3S-NH2.

#### Figure S66: ^1^H NMR of C18-3S-Cy3.
